# Identification of Medicinal *Bidens* Plants for Quality Control Based on Organelle Genomes

**DOI:** 10.3389/fphar.2022.842131

**Published:** 2022-02-14

**Authors:** Liwei Wu, Liping Nie, Shiying Guo, Qing Wang, Zhengjun Wu, Yulin Lin, Yu Wang, Baoli Li, Ting Gao, Hui Yao

**Affiliations:** ^1^ National Engineering Laboratory for Breeding of Endangered Medicinal Materials, Institute of Medicinal Plant Development, Chinese Academy of Medical Sciences and Peking Union Medical College, Beijing, China; ^2^ China Resources Sanjiu Medical & Pharmaceutical Co., Ltd, Shenzhen, China; ^3^ Key Laboratory of Plant Biotechnology in Universities of Shandong Province, College of Life Sciences, Qingdao Agricultural University, Qingdao, China; ^4^ Engineering Research Center of Chinese Medicine Resources, Ministry of Education, Beijing, China

**Keywords:** *bidens*, species identification, super barcode, organelle genomes, quality control

## Abstract

*Bidens* plants are annuals or perennials of Asteraceae and usually used as medicinal materials in China. They are difficult to identify by using traditional identification methods because they have similar morphologies and chemical components. Universal DNA barcodes also cannot identify *Bidens* species effectively. This situation seriously hinders the development of medicinal *Bidens* plants. Therefore, developing an accurate and effective method for identifying medicinal *Bidens* plants is urgently needed. The present study aims to use phylogenomic approaches based on organelle genomes to address the confusing relationships of medicinal *Bidens* plants. Illumina sequencing was used to sequence 12 chloroplast and eight mitochondrial genomes of five species and one variety of *Bidens*. The complete organelle genomes were assembled, annotated and analysed. Phylogenetic trees were constructed on the basis of the organelle genomes and highly variable regions. The organelle genomes of these *Bidens* species had a conserved gene content and codon usage. The 12 chloroplast genomes of the *Bidens* species were 150,489 bp to 151,635 bp in length. The lengths of the eight mitochondrial genomes varied from each other. Bioinformatics analysis revealed the presence of 50–71 simple sequence repeats and 46–181 long repeats in the organelle genomes. By combining the results of mVISTA and nucleotide diversity analyses, seven candidate highly variable regions in the chloroplast genomes were screened for species identification and relationship studies. Comparison with the complete mitochondrial genomes and common protein-coding genes shared by each organelle genome revealed that the complete chloroplast genomes had the highest discriminatory power for *Bidens* species and thus could be used as a super barcode to authenticate *Bidens* species accurately. In addition, the screened highly variable region *trnS-GGA-rps4* could be also used as a potential specific barcode to identify *Bidens* species.

## 1 Introduction

*Bidens* plants are annuals or perennials of Asteraceae. In China, this genus includes 10 species (Shi et al., 2011), five of which are medicinal plants recorded in different local standards for Chinese medicinal materials. *Bidens* plants have a long history of medicinal use in China (Chen et al., 2009). There are many kinds of compounds in the *Bidens* plants, including flavonoids, phenylpropanoids, triterpenoids, alkaloids and organic acids, of which flavonoids are the main effective components in the medicinal *Bidens* plants (Wang et al., 2010). Modern pharmacological studies show that these compounds in *Bidens* plants have antiinflammatory, analgesic, antibacterial, antitumor, hypolipidemic, and liver protection functions (Lin et al., 2013; Shandukani et al., 2018). However, some problems exist in the records of these medicinal plants in local standards. For example, their scientific names are inconsistent with their Chinese names and records in the Flora of China. *Bidens* plants commonly have homonyms and synonyms. Furthermore, they are difficult to identify by using traditional identification methods because they have similar morphologies and chemical components (Bartolome et al., 2013; Chen et al., 2013; Wang et al., 2014; Yang et al., 2020). Reports on the molecular identification of *Bidens* species are limited. Tsai et al. (2008) (Tsai et al., 2008) used the noncoding regions of the chloroplast genome (*trnL* intorn and *trnL-trnF*) and nuclear ribosomal DNA (ITS1, 5.8S and ITS2) to identify *Bidens* species and found that ITS1, 5.8S, ITS2 and the *trnL* intron could separate only *Bidens biternata* and *B. pilosa* var. *pilosa* from each other. Our preliminary experiments showed that the universal DNA barcodes ITS, ITS2 and *psbA-trnH* were all ineffective in identifying *Bidens* species. The difficulties encountered in the identification of *Bidens* species seriously hinder the development of medicinal *Bidens* plants and reduce their medicinal quality. An accurate and effective method for identifying medicinal *Bidens* plants is urgently needed.

The main content of phylogenomic studies includes the use of large-scale molecular data to investigate the phylogenetic relationships between organisms at the genomic level and the application of evolutionary relationships to study the evolutionary mechanisms of genomes, such as the process of DNA repair and the functional annotation of unknown genes (Delsuc et al., 2005). In general, plant cells contain three kinds of genomes, namely, the chloroplast, mitochondrial and nuclear genomes. The chloroplast and mitochondrial genomes are also called organelle genomes. The relative abundances of the three genomic DNA in cells show significant differences. For example, a leaf cell of the model plant *Arabidopsis thaliana* contains approximately 1,000 copies of chloroplast DNA, 100 copies of mitochondrial DNA and two copies of nuclear genomic DNA (Logan 2006). Given the important role of the chloroplast and mitochondrial genomes in phylogenetic and nucleo-cytoplasmic interactions, their sequence analysis is becoming increasingly important. The chloroplast is an organelle that plays an important role in plant photosynthesis (Clegg et al., 1994). The chloroplast genome is more conserved than the nuclear genome in terms of gene content and order and contains more variations than DNA barcodes. Therefore, the chloroplast genome is widely used in species identification and plant evolution studies. Zhang et al. (Zhang et al., 2019) found that the whole chloroplast genome could be used as a super barcode to identify *Dracaena* species. Chen et al. (Chen et al., 2018; Chen et al., 2019) successfully identified six *Ligularia* and three *Ephedra* herbs by using the chloroplast genome as a super barcode. A growing number of works have provided support showing that identifying related species by using the molecular markers of the chloroplast genome or the complete chloroplast genome as super barcodes is practicable. The mitochondrial genome, another important organelle genome, is usually similar to the chloroplast genome, which has a circular molecular structure. The mitochondrial genome is used in species identification because it is complex and highly variable with abundant noncoding regions and introns and a relatively fixed sequence (An et al., 2017; Park and Lee, 2020; Jeong and Lee, 2021). The mitochondrial genome of angiosperms could reveal the phylogenetic relationship between species and be used to investigate intraspecific differentiation (Fujii et al., 2010). However, the number of reported mitochondrial genomes is not as large as that of chloroplast genomes.

Studying organelle genomes is an essential way to analyse the genetic information of a species. The excavation of organelle genomes is helpful for analysing the inherent properties and changes of organelle genomes and thus contributes to the genetic evolution, identification and breeding research of various species. In addition, organelle genomes are important data sources for comparative genomics, phylogenetics and population genetics (Qian 2014). Organelle genomes are smaller and easier to sequence than nuclear genomes. In this work, we sequenced and analysed the 12 chloroplast and eight mitochondrial genomes of five species and one variety of *Bidens* to solve the difficulties encountered in the identification of *Bidens* species. Finally, we constructed phylogenetic trees by using different datasets and analysed the feasibility of identifying *Bidens* species on the basis of organelle genomes. This research could provide a foundation for the species identification, phylogeny, medication safety and plant resource protection of *Bidens*.

## 2 Materials and Methods

### 2.1 DNA Sources

The DNA sources were the fresh leaves of *B. biternata*, *B. bipinnata*, *B. pilosa* var. *pilosa*, *B. pilosa* var. *radiata*, *B. parviflora* and *B. tripartita*. These species were identified by Prof. Yulin Lin from the Institute of Medicinal Plant Development (IMPLAD), Chinese Academy of Medical Sciences and Peking Union Medical College. Voucher specimens were deposited in the herbarium at IMPLAD. The ID numbers and collecting locations are shown in Supplementary Table S1. The total DNA of the species was extracted by using the DNeasy Plant Mini Kit (Qiagen Co., Germany), and DNA concentration and quality were assessed by using Nanodrop 2000C spectrophotometry and electrophoresis in 1% (w/v) agarose gel, respectively.

### 2.2 DNA Sequencing, Assembly and Annotation

The DNA was used to generate libraries with an average insert size of 350 bp and sequenced by using Illumina NovaSeq6000 in accordance with standard protocols, and the sequencing information was shown in Supplementary Tables S2, S3. Paired-end sequencing was performed to obtain 150 bp sequences at both ends of each molecule. Adapters and low-quality regions in the original data were trimmed by applying Trimmomatic software (Bolger et al., 2014). Reference organelle sequences from the family of Asteraceae were downloaded from NCBI genome resources (https://www.ncbi.nlm.nih.gov/genome). The gene sequences of each reference were extracted to build a custom database, and clean reads were mapped by using BWA 0.7.17 (Li and Durbin 2009). NOVOPlasty 4.2 (Dierckxsens et al., 2017) was used to assemble organelle genomes, which needed a sequence as the initial seed. For the chloroplasts, a read from the *psbA* gene was selected as the seed input, and the output chloroplast sequences were manually adjusted for the start position. For the mitochondria, seed reads from the conserved *cox* and *nad* genes were separately tested, and the output contigs were combined and manually linked to obtain the mitochondrial sequences. Then, clean reads were mapped back to the chloroplast and mitochondrial genomes and inspected in IGV (Robinson et al., 2017) to exclude any assembly error. Finally, a custom-made script that took the assembly and the bam files as input was utilised to correct ambiguous bases and generate the complete organelle genome sequences. The sequences were initially annotated by using the CPGAVAS2 software (Shi et al., 2019) and the GeSeq (Tillich et al., 2017) and corrected manually. tRNAs were annotated by using tRNAscan-SE software (Schattner et al., 2005). Genes, introns and coding region boundaries were compared with reference sequences. Then the borders of LSC, SSC and IR regions in the chloroplast genomes were validated by designing primers and polymerase chain reaction (Supplementary Table S4).

### 2.3 Structural Analyses

Chloroplast and mitochondrial genome maps were generated by using the Organellar Genome DRAW v1.2 (Lohse et al., 2007) and manually corrected. The CodonW software (Sharp and Li 1987) was adopted to analyse codon usage. Simple sequence repeats (SSRs) were detected by using the MISA (Beier et al., 2017) with the definition of ≥ 10 repeat units for mononucleotide SSRs, ≥ 5 repeat units for dinucleotide SSRs, ≥ 4 repeat units for trinucleotide SSRs, and ≥ 3 repeat units for tetranucleotide, pentanucleotide and hexanucleotide SSRs. Long repeated sequences were detected by using REPuter (Kurtz et al., 2001). Sequence homology analysis was carried out by using EMBOSS (Rice et al., 2000). The boundaries of the four regions of the chloroplast genomes were compared by applying IRscope (Amiryousefi et al., 2018).

### 2.4 Comparative and Phylogenetic Analyses

The chloroplast genomes of *Bidens* species were compared by using mVISTA software (Frazer et al., 2004). The nucleotide diversity (Pi) values of shared genes and intergenic spacers were calculated with DnaSP software (Librado and Rozas 2009). By combining the mVISTA results and Pi values, seven highly variable regions were screened out. The complete organelle genome sequences of Asteraceae species and common protein-coding genes shared by these genomes were used to construct maximum likelihood (ML) phylogenetic trees by utilising IQ-TREE (Nguyen et al., 2015) with a bootstrap of 1,000 repetitions. ML analysis was conducted based on the TVM + F + R4 (complete chloroplast genomes), TVM + F + I + G4 (complete mitochondrial genomes), TVM + F + R3 (common protein-coding genes shared by chloroplast genomes), and GTR + F + G4 (common protein-coding genes shared by mitochondrial genomes) models. MEGA software (Tamura et al., 2013) was used to construct Neighbor-joining (NJ) phylogenetic trees based on seven highly variable regions. NJ analysis was conducted based on the K2P model.

## 3 Results

### 3.1 Organelle Genome Structure of *Bidens* Species

**FIGURE 1:**
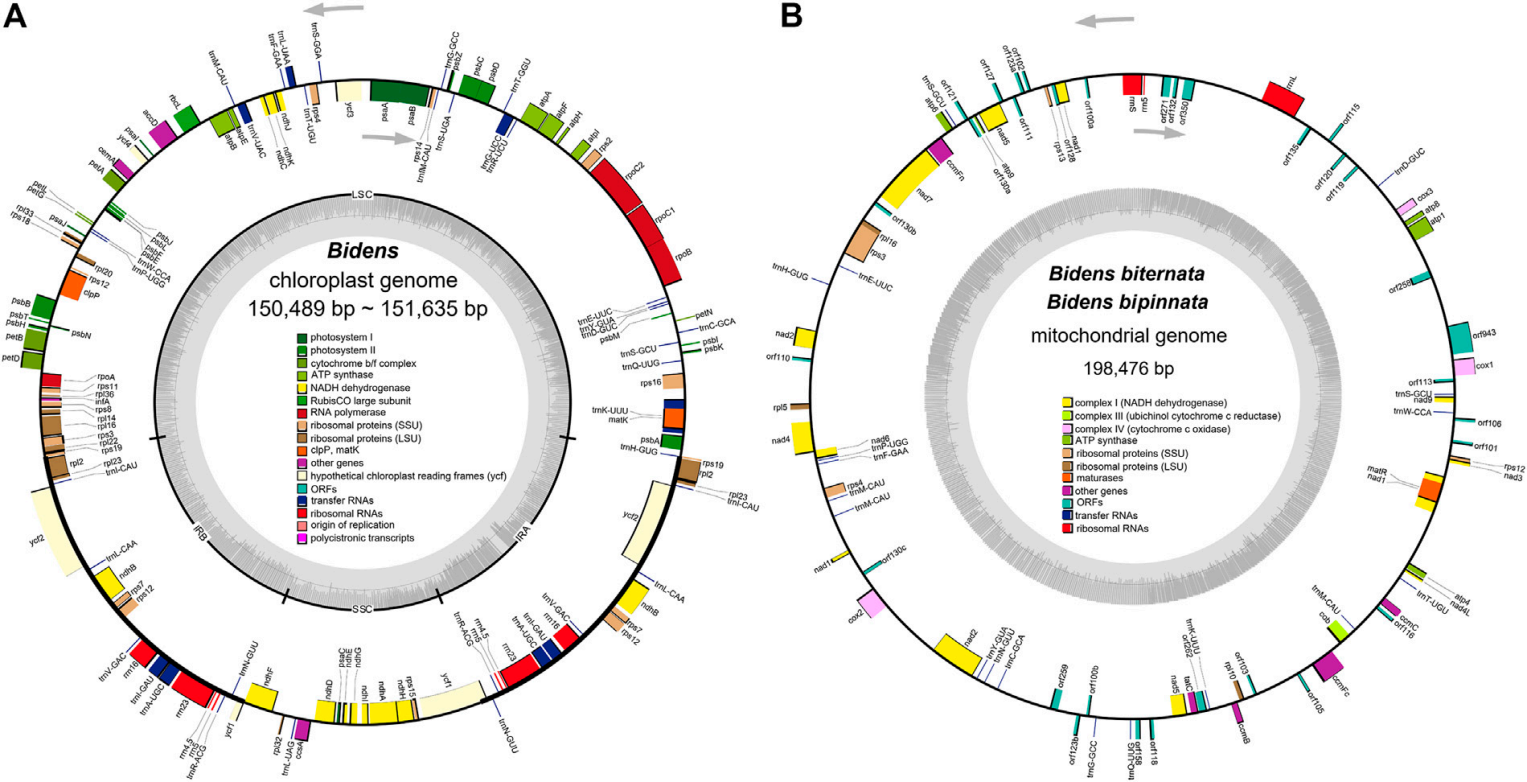
Chloroplast **(A)** and mitochondrial **(B)** genome maps of *Bidens* species. The transcription directions of the inner and outer genes are listed clockwise and anticlockwise, respectively, and are represented by arrows. The dark grey colour in the inner circle corresponds to the GC content, whereas the light grey colour corresponds to the AT content.

The 12 chloroplast genomes of these *Bidens* species showed a typical circular tetramerous structure and included two inverted repeats (IRs), a large single copy (LSC) and a small single copy (SSC) (Figure 1A). The lengths of the chloroplast genomes of the same species were the same or similar. The total lengths of the chloroplast genomes were 150,489 bp (*B. tripartita*) to 151,635 bp (*B. pilosa* var. *radiata*). The sizes of the LSC regions ranged from 83,499 bp (*B. tripartita*) to 83,899 bp (*B. biternata*). The sizes of the SSC regions varied between 17,628 bp (*B. tripartita*) and 18,439 bp (*B. pilosa* var. *radiata*). The sizes of the IR regions varied from 24,652 bp (*B. bipinnata*) to 24,701 bp (*B. pilosa* var. *radiata*). The total GC contents of the 12 chloroplast genomes were all 37.5%, indicating that the base composition of the *Bidens* species was relatively conserved (Supplementary Table S5).

**FIGURE 2:**
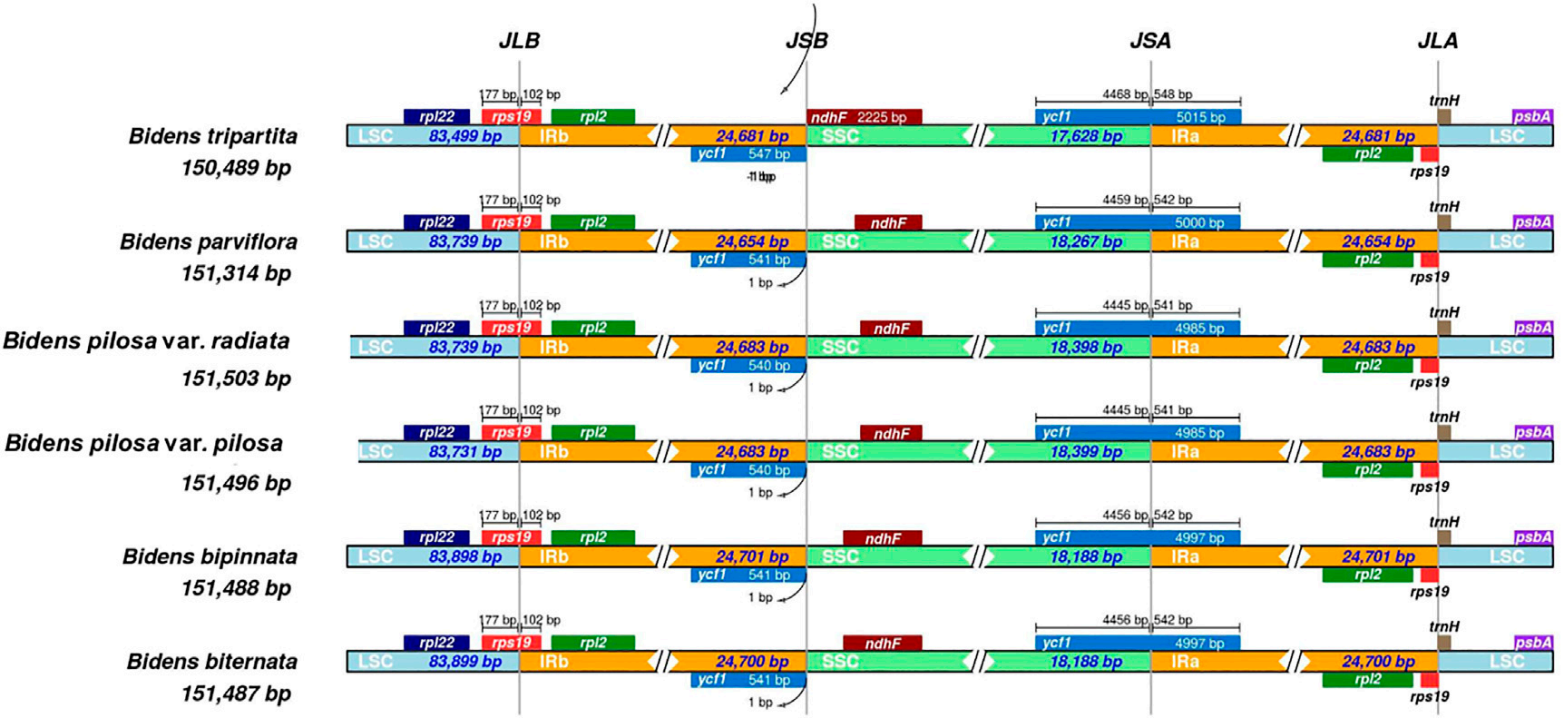
Comparison amongst the borders of LSC, SSC and IR regions in the chloroplast genomes of *Bidens* species. The number above the gene features indicates the distance between the ends of genes and the border sites. These features are not to scale. JLB: junction of LSC/IRb; JSB: junction of IRb/SSC; JSA: junction of SSC/IRa; JLA: junction of IRa/LSC.

The 12 chloroplast genomes were all annotated with 130 genes, including 85 protein-coding genes, 37 tRNA genes and eight rRNA genes (Supplementary Table S6). Amongst these genes, 17 were located in IR regions. They included six protein-coding genes (*ndhB*, *rpl2*, *rpl23*, *rps7*, *rps12* and *ycf2*), four rRNA genes (*rrn4*, *rrn4.5*, *rrn5* and *rrn16*) and seven tRNA genes (*trnA-UGC*, *trnI-CAU*, *trnI-GAU*, *trnL-CAA*, *trnN-GUU*, *trnR-ACG* and *trnV-GAC*). In addition, *ycf1* and *rps19* were annotated as pseudogenes. At the junctions, the gene positions in the boundary regions of the chloroplast genomes of the *Bidens* species were conserved. The difference was that the *ndhF* gene of *B. tripartita* was located at the boundary of the SSC and IRb regions, and the *ndhF* genes of the other five species were located in the SSC regions (Figure 2). Coding regions (protein-coding regions, tRNA genes and rRNA genes) accounted for 56.0–59.4% of the regions, and the rest were noncoding regions (pseudogenes, introns and gene spacers).

The eight mitochondrial genomes of these *Bidens* species all had a circular molecular structure (Figure 1B; Supplementary Figure S1) that ranged in length from 183 kb (*B. pilosa* var. *pilosa*) to 216 kb (*B. tripartita*). The GC contents were between 45.4 and 45.8%. The mitochondrial genomes of *B. biternata* and *B. bipinnata* were annotated with 83 genes, including 30 protein-coding genes, 18 tRNA genes, three rRNA genes and 32 open reading frames (ORFs). A total of 81, 87, 90 and 82 genes were annotated in the mitochondrial genomes of *B. pilosa* var. *pilosa*, *B. pilosa* var. *radiata*, *B. parviflora* and *B. tripartita*, respectively. The total proportion of all genes in the mitochondrial genomes of the *Bidens* species was between 24.4 and 28.6% (Supplementary Table S7).

The genes annotated in the mitochondrial genomes of the *Bidens* species could be divided into 11 categories (Supplementary Table S8). The similar gene types and numbers of complex I, complex III, complex IV, complex V, rRNA genes and maturation enzyme genes indicated that these genes were highly conserved in the mitochondrial genomes. The number of tRNA genes ranged from 18 to 20, and the number of ORFs was quite different.

### 3.2 Relative Synonymous Codon Usage

**FIGURE 3:**
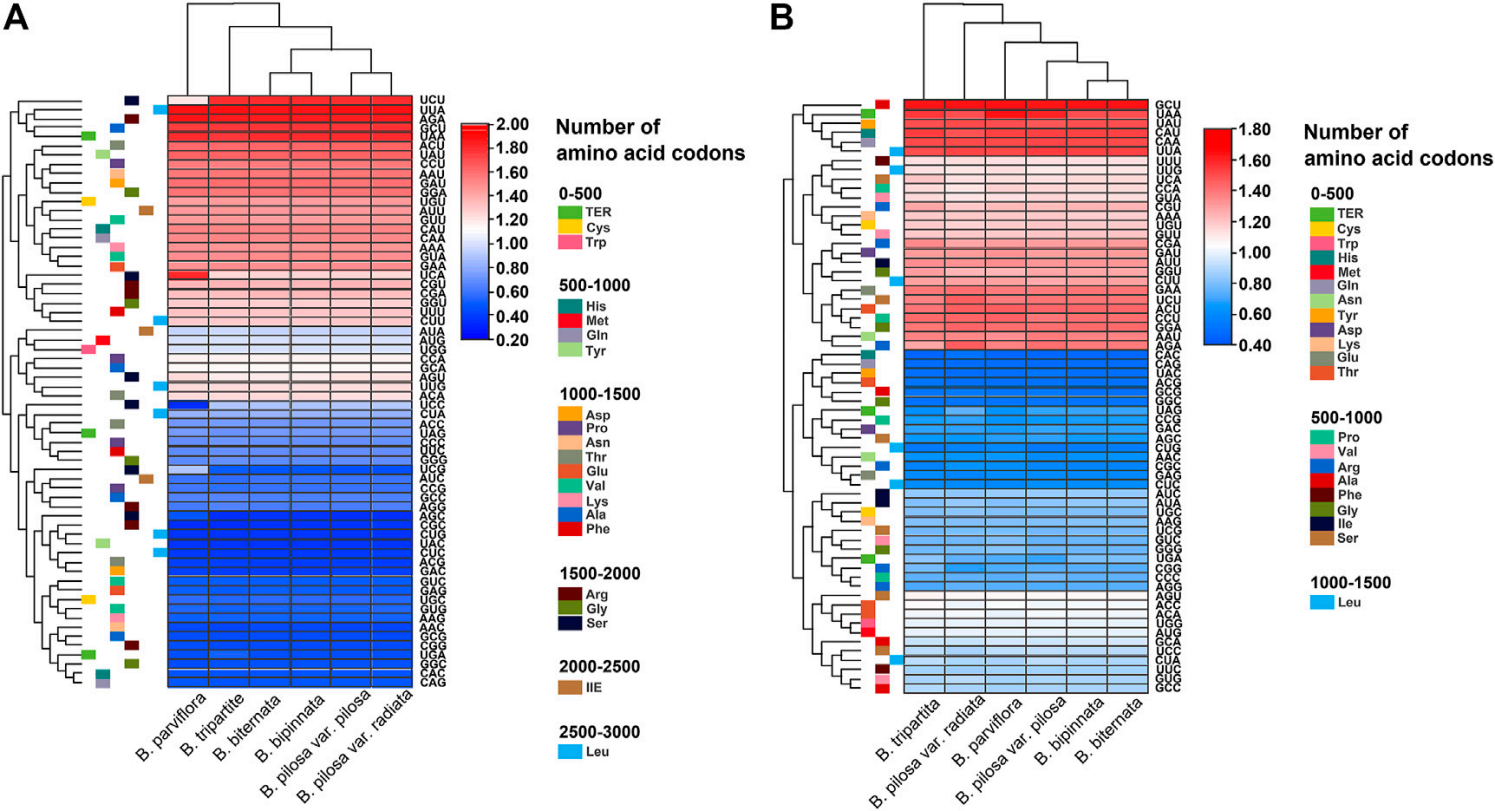
Heat map of the RSCU values of the chloroplast **(A)** and mitochondrial **(B)** genomes of *Bidens* species.

The relative synonymous codon usage (RSCU) of the chloroplast and mitochondrial genomes of the *Bidens* species was calculated on the basis of all protein-coding genes (Figure 3). The results showed that the chloroplast and mitochondrial genomes of the *Bidens* species contained 64 types of codons encoding 20 amino acids. Leucine, serine and arginine all had six types of codons. Amongst all amino acids, leucine had the highest number of codons, whereas cysteine had the lowest.

Given that methionine and tryptophan possess only one codon each, no codon usage bias was found, and the RSCU value was 1. Codon usage bias was found for the rest of the amino acid codons. In the chloroplast genomes, 30 codons were found with RSCU > 1, of which 29 were A/U-ending codons, and 34 codons were found with RSCU ≤ 1, of which 31 were G/C-ending codons. The highest and lowest RSCU values were recorded for UUA and CGC, which encoded leucine and arginine, respectively. In the mitochondrial genomes, 30 codons were found with RSCU > 1, of which 28 were A/U-ending codons, and 34 codons were found with RSCU ≤ 1, of which 30 were G/C-ending codons. The highest and lowest RSCU values were recorded for GCU and CAC, which encoded alanine and histidine, respectively. These results indicated that the chloroplast and mitochondrial genomes exhibited a higher bias towards A/U-ending codons than towards G/C-ending codons.

### 3.3 Simple Sequence Repeats and Long Repetitive Sequences in the Organelle Genomes of *Bidens* Species

In this study, a total of 56 (*B. parviflora*) to 71 (*B. tripartita*) SSRs were detected in the chloroplast genomes of these *Bidens* species. The distribution of SSRs in the three samples of *B. biternata* was the same. It was also the same in the four samples of *B. bipinnata*. *Bidens bipinnata* had one more mononucleotide repeat than *B. biternata*, and the remaining SSR types and numbers were the same. SSR distribution differed between the two *B. pilosa* var. *radiata* samples. The MW551955 sample had two more mononucleotide repeats, one more dinucleotide repeat and one more trinucleotide repeat than the MW551952 sample, indicating obvious SSR polymorphism in the chloroplast genome of *B. pilosa* var. *radiata*. Mononucleotide to hexanucleotide repeats were present in the *Bidens* species, most of which were mononucleotide repeats, followed by dinucleotide and tetranucleotide repeats (Supplementary Table S9). The SSRs of the mitochondrial genomes of the *Bidens* species were also analysed. A total of 50 (*B. pilosa* var. *pilosa*) to 62 (*B. pilosa* var. *radiata*) SSRs were detected in the mitochondrial genomes of the *Bidens* species. Six types of SSR were detected in the mitochondrial genomes of *B. biternata*, *B. bipinnata*, *B. pilosa* var. *radiata* and *B. parviflora*, whereas only five types were detected in *B. pilosa* var. *pilosa* and *B. tripartita* (Supplementary Table S10).

Some repetitive sequences with length ≥ 30 bp and sequence similarity ≥ 90% were found. These sequences were called long repetitive sequences, namely, forward (F), palindrome (P), reverse (R) and complement (C). In the chloroplast genomes of *Bidens* species, our analysis revealed 46 (*B. biternata* and *B. bipinnata*) to 114 (*B. pilosa* var. *radiata*) long repeats, most of which were F and P repeats. The long repeats mainly had lengths of 30–39 bp and included four types. Repeats that were larger than 40 bp were mainly F and P repeats. No repeats with lengths of 50–59 and 60–69 bp were found in *B. biternata*, *B. bipinnata* and *B. parviflora*, and no repeat with lengths of 60–69 bp was found in *B. tripartita* (Supplementary Table S11). The mitochondrial genomes of *Bidens* species all contained F and P repeats. The mitochondrial genomes of *B. biternata*, *B. bipinnata*, *B. pilosa* var. *pilosa*, *B. pilosa* var. *radiata* and *B. parviflora* contained R repeats. Only the mitochondrial genomes of *B. biternata*, *B. bipinnata* and *B. pilosa* var. *pilosa* contained C repeats. The mitochondrial genome of *B. tripartita* did not contain R and C repeats. The F and P repeats accounted for more than 90% of the long repeats in the mitochondrial genomes of the *Bidens* species. The number of long repeats was the highest in *B. pilosa* var. *radiata* and *B. tripartita*, with 106 and 108 F repeats, respectively. In these species, the number of repeats with lengths of 30–39 bp was the largest, followed by that of repeats with lengths of 40–99 bp; repeats with length ≥ 1 kb were rare (Supplementary Table S12).

### 3.4 Variation in the Organelle Genomes of *Bidens* Species

**FIGURE 4:**
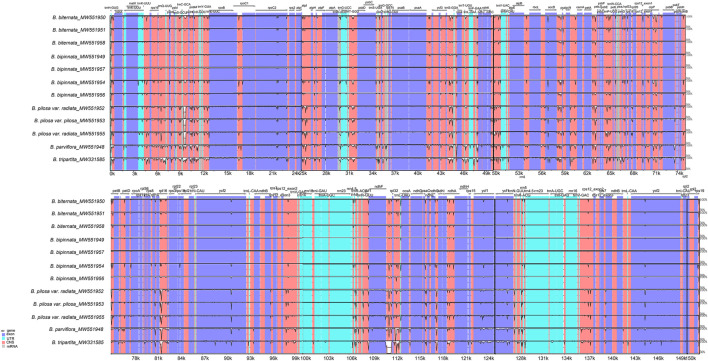
Global alignment of the chloroplast genomes of *Bidens* species. The *x*-axis represents the coordinates in the chloroplast genome. The *y*-axis indicates the average percent identity of sequence similarity, which ranged between 50 and 100%, in the aligned regions.

Consistent with the chloroplast genome analysis above, the global comparison of the chloroplast genomes of the *Bidens* species showed that the seven chloroplast genomes of *B. biternata* and *B. bipinnata* were highly similar. A high degree of similarity was found between the samples of *B. pilosa* var. *pilosa* and *B. pilosa* var. *radiata*. The difference between *B. tripartita* and the other five species was the largest, and a mutation locus was found in the intergene region of *ndhF-rpl32*. In addition, the results revealed that the variation in the noncoding region was considerably greater than that in the coding region. Most of the variation was located in the LSC and SSC regions, and slight variation occurred in the IR regions. The rRNA genes of these species were highly conserved with little variation (Figure 4).

**FIGURE 5:**
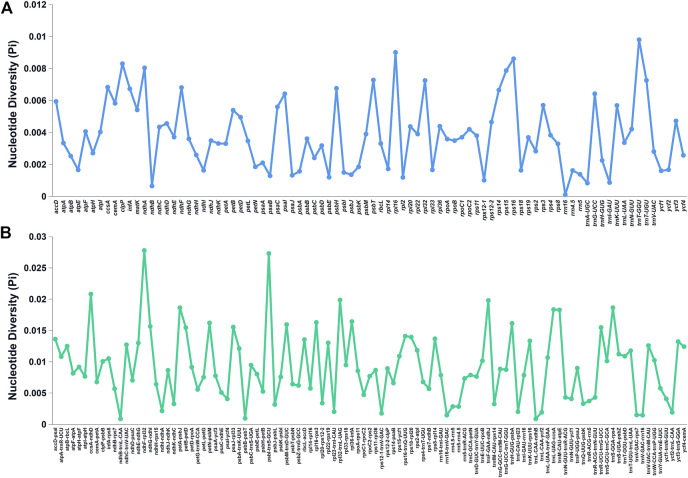
Nucleotide diversity of various shared regions in the chloroplast genomes of *Bidens* species. **(A)** Pi values in the gene regions. **(B)** Pi values in the intergenic spacer regions.

The Pi values of the shared genes and intergenic spacers of the chloroplast genomes of the *Bidens* species were calculated. Figure 5 shows the intergenic spacers and genes with Pi > 0. Intergenic spacers had more polymorphisms than gene regions, and these results were consistent with the mVISTA analysis results. By combining the mVISTA and Pi results, seven candidate highly variable regions (Pi > 0.018; length > 200 bp) were screened out for species identification and relationship studies.

**FIGURE 6:**
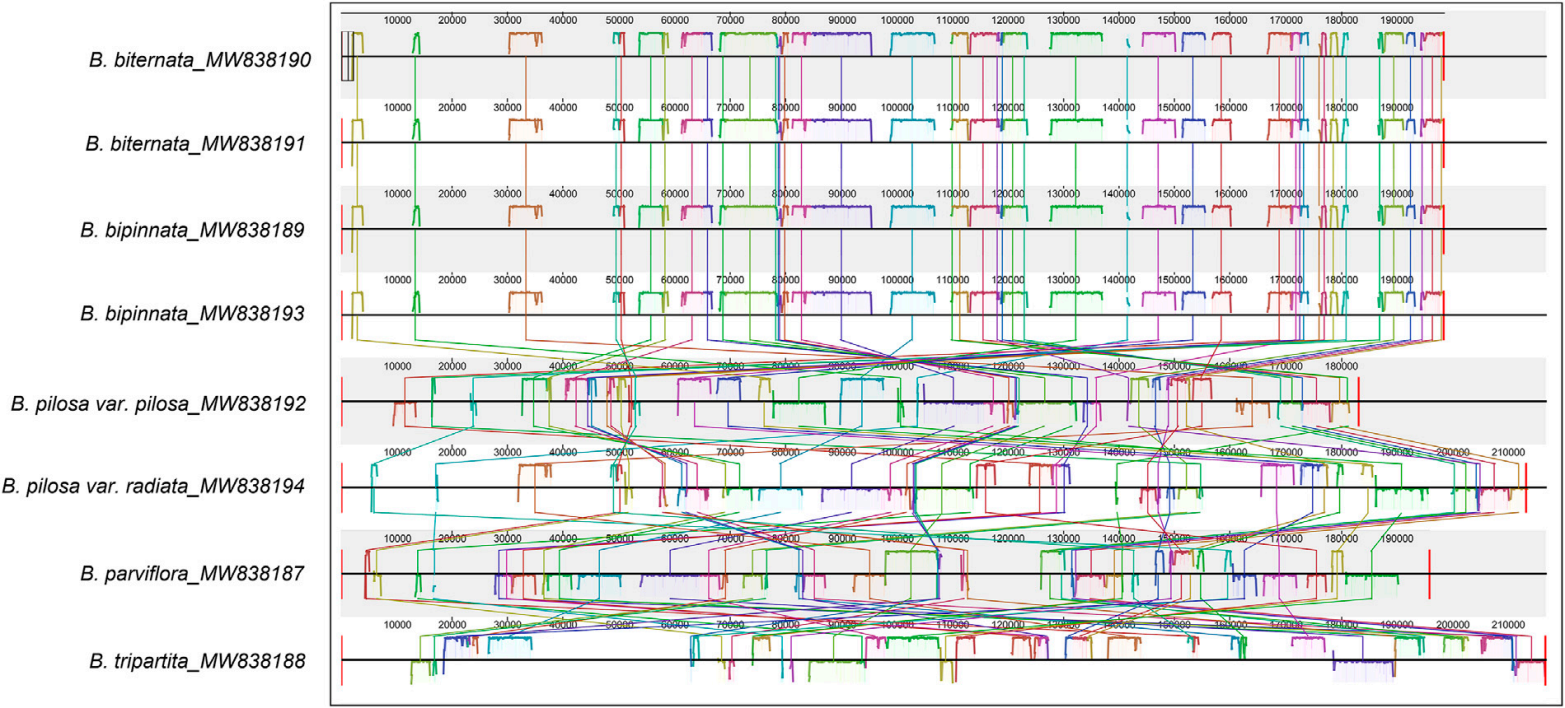
Collinearity analysis of the chloroplast genomes of *Bidens* species. Local collinear blocks are represented by blocks of the same colour connected by lines.

Consistent with the result based on chloroplast genomes, the results of collinearity and homology analysis showed that the mitochondrial genomes of *B. biternata* and *B. bipinnata* had high homology. The mitochondrial genomes of *B. pilosa* var. *pilosa*, *B. pilosa* var. *radiata*, *B. parviflora* and *B. tripartita* were quite different from those of *B. biternata* and *B. bipinnata* (Figure 6). The dot plot of the mitochondrial genome sequences between *B. biternata* and *B. bipinnata* showed an evident line on the diagonal, indicating that the chloroplast genome sequences of *B. biternata* and *B. bipinnata* had high homology (Supplementary Figure S2). In the dot plot of mitochondrial genome sequences between *B. pilosa* var. *pilosa* and *B. pilosa* var. *radiata*, only several diagonal lines made up of marker points were parallel to the diagonal lines, which represent the same substring of two sequences. This result indicated that the mitochondrial genome sequences of *B. pilosa* var. *pilosa* and *B. pilosa* var. *radiata* have low homology (Supplementary Figure S3).

### 3.5 Identification and Phylogenetic Analysis of *Bidens* Species

**FIGURE 7:**
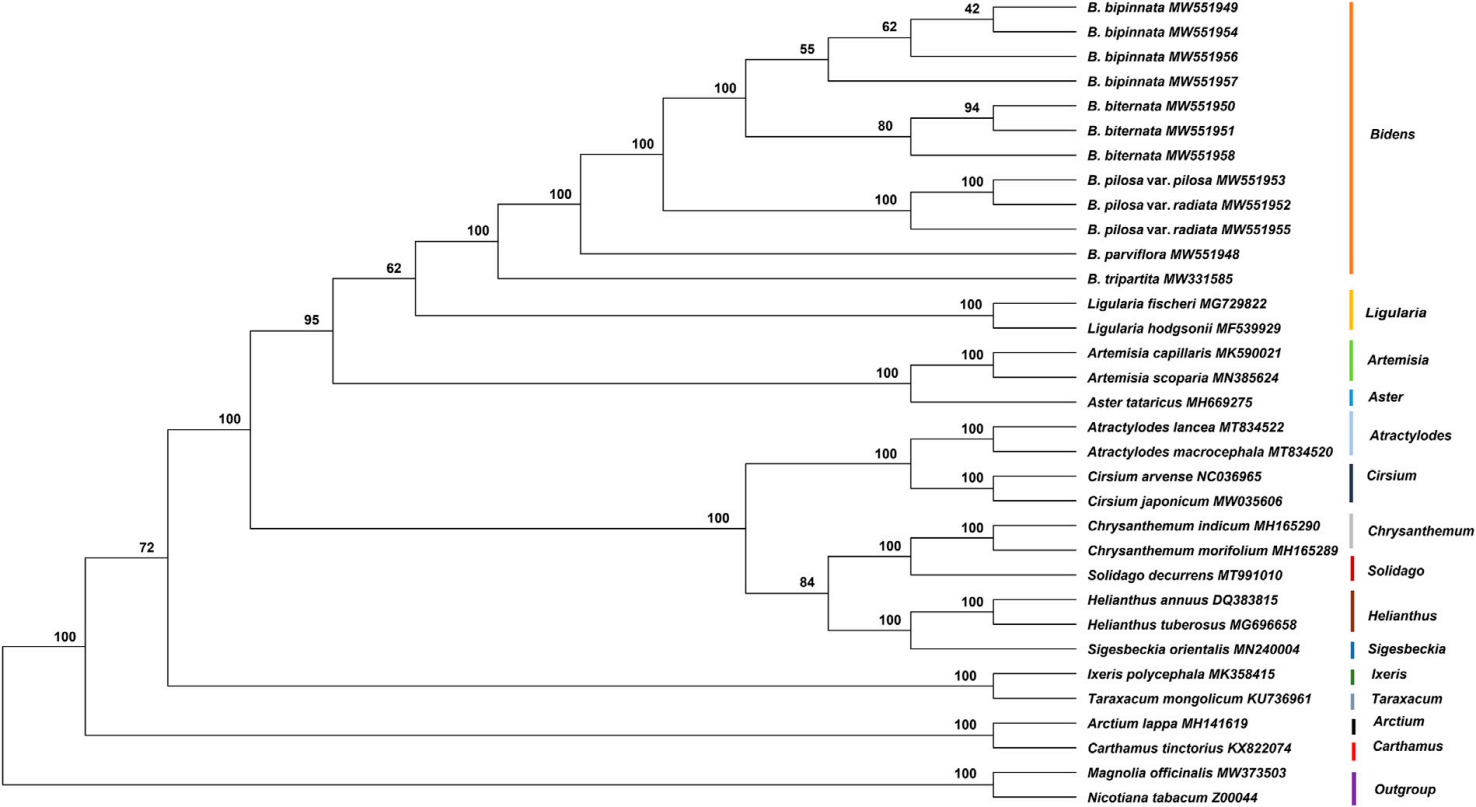
Phylogenetic tree constructed by using the ML method based on the complete chloroplast genome sequences of Asteraceae species. The numbers at the nodes are the values for bootstrap support.

In the current study, the complete chloroplast genome sequences of five species and one variety of *Bidens* and 21 other Asteraceae species were used to construct a phylogenetic tree with *Magnolia officinalis* and *Nicotiana tabacum* as the outgroups (Figure 7). The results showed that the *Bidens* species clustered in a big branch and that different samples of the same species clustered together. The three samples of *B. biternata* clustered in one branch, which was sister to the cluster comprising the four samples of *B. bipinnata*. *Bidens pilosa* var. *radiata* and *B. pilosa* var. *pilosa* clustered together. *Bidens tripartita* and *B. parviflora* clustered in a single branch, respectively. In addition, the species in *Ligularia* were close to those in *Bidens*.

Then, the common protein-coding genes shared in these chloroplast genomes were applied to construct the ML phylogenetic tree (Supplementary Figure S4). The result was slightly different from the finding based on the complete chloroplast genome sequences. The four samples of *B. bipinnata* did not cluster together. For the seven candidate highly variable regions, only *trnS-GGA-rps4* showed the capability to identify *Bidens* species (Supplementary Figure S5). The species of *B. bipinnata*, *B. biternata* and *B. pilosa* clustered in different branches, respectively. In contrast to the tree based on the complete chloroplast genome, *B. parviflora* was sister to the *B. pilosa* species but with a low bootstrap value.

The ML phylogenetic tree was constructed by using the complete mitochondrial genomes and common protein-coding genes shared by the mitochondrial genomes of Asteraceae species, including the *Bidens* species (Supplementary Figures S6, S7). The *Bidens* species did not cluster together in the tree based on the complete mitochondrial genomes, whereas the *Bidens* species clustered together in the tree based on the common protein-coding genes shared by these mitochondrial genomes. However, species within the genus *Bidens* were indistinguishable from each other. The results showed that in contrast to the chloroplast genome, the mitochondrial genome was unsuitable for the identification and phylogenetic analysis of *Bidens* species.

## 4 Discussion

The lengths of the chloroplast genomes of these *Bidens* species were similar to those of other reported Asteraceae species, such as *Ligularia* species (151,118–151,253 bp) (Chen et al., 2018), *Artemisia frigida* (151,076 bp) (Liu et al., 2013) and *Stilpnolepis centiflora* (151,017 bp) (Shi and Xie 2020). The GC contents of the chloroplast genomes of the *Bidens* species were all 37.5%, which was similar to those of *Carthamus tinctorius* (Lu et al., 2016), *Saussurea involucrata* (Xie et al., 2017) and *Arctium lappa* (Xing et al., 2019). The majority of the chloroplast genomes of Asteraceae species, such as *Soroseris umbrella* (Lv et al., 2020), *Saussurea inversa* and *Saussurea medusa* (Wang et al., 2021), as well as *Pertya phylicoides* (Wang et al., 2020), contained approximately 130 genes, which included approximately 113 unique genes. The global comparison of the chloroplast genomes showed that the variation of the noncoding region was considerably larger than that of the coding region, most of the variation was located in the LSC and SSC regions, and very little variation occurred in the IR regions. The same findings were also found for *Arctium lappa* (Nie et al., 2020), *Ligularia* species (Chen et al., 2018) and *Artemisia* species (Liu et al., 2013). Moreover, we compared the CDS of the chloroplast genomes of 11 Asteraceae medicinal plants, including the *Bidens* species in this study, *Bidens frondose* (Knope et al., 2020), *Arctium lappa* (Nie et al., 2020), *Atractylodes lancea* (Shi et al., 2021), *Aster tataricus* (Shen et al., 2018) and *Artemisia annua* (Shen et al., 2017). The results showed that although the gene composition of the chloroplast genome of Asteraceae medicinal plants was highly conserved, slight variations were still present. The chloroplast genome of *A. annua*, but not the chloroplast genomes of other 10 medicinal plants, contained the photosystem II gene *psbG*. Only the chloroplast genome of *B. frondose* did not contain the *ycf1* gene. The chloroplast genomes of 11 medicinal plants contained the *ycf2* gene, whereas in the chloroplast genome of *A. tataricus*, *ycf2* gene did not repeat in the IR regions. The *ycf15* gene existed only in the chloroplast genome of *B. frondose* and *A. annua* and not in other medicinal plants (Supplementary Figure S8). Bioinformatics analysis revealed the presence of SSRs and long repeats in the chloroplast genome of *Bidens* species. Given that long repeats are abundant in the chloroplast genomes of some highly recombinant algae and angiosperms, especially at the end of the recombinant site, they are considered to be one of the main reasons for promoting the recombination of chloroplast genomes. However, in the chloroplast genome without recombination, the role of these repeats remains unclear (Qian 2014). The SSRs and long repeats contained in the chloroplast genome can be used as important sources of molecular markers for the development of research on *Bidens* species.

The lengths of the mitochondrial genomes of these *Bidens* species were similar to those of most land plants reported in the organelle database of NCBI (Supplementary Figure S9) and was similar to that of *Tanacetum vulgare* (Won et al., 2018). The total GC content of the mitochondrial genomes of the *Bidens* species was between 45.4 and 45.8% and were similar to that of *Helianthus annuus* (Grassa et al., 2016). The number of CDS in the mitochondrial genomes of the *Bidens* species was approximately 30 genes and was similar to that of most of reported plants in the NCBI organelle genome databases (30–45). In contrast to those of the conserved chloroplast genome, the sizes and structures of the mitochondrial genomes of angiosperms vary greatly (Levings and Brown 1989; Adams et al., 2002). Studies have shown that the plant mitochondrial genome is a mixture of DNA molecules with different shapes (Kubo and Newton 2008). The mitochondrial genomes of chrysanthemum and sunflower are circular (Makarenko et al., 2019; Wynn and Christensen 2019), whereas those of wheat, rape and cucumber comprise multiple rings (Handa 2003; Ogihara et al., 2005; Alverson et al., 2011). The mitochondrial genomes of plants are considerably larger than those of animals and range from 200 to 2,500 kb with a variation of more than 10 times (Levings and Brown 1989). In most angiosperms, the size of the mitochondrial genome is concentrated within the range of 300–600 kb (Levings and Brown 1989). The secondary structure of the mitochondrial genome is complex and changeable, and gene recombination frequently occurs in the mitochondrial genomes of angiosperms (Kubo and Newton 2008; Gualberto and Newton 2017). Mitochondrial genomes are generally used for high-level classification, such as intergenus and interfamily classification (Qiu et al., 2010).

In this study, phylogenetic trees were constructed on the basis of the complete chloroplast genomes, complete mitochondrial genomes, common protein-coding genes shared by each organelle genome, and seven selected highly variable regions. The complete chloroplast genomes showed the best capability for the identification and phylogenetic analysis of *Bidens* species. The chloroplast genome is an ideal material for species authentication and phylogenetic studies because it can be maternally inherited, usually does not undergo genetic recombination and has highly conserved gene content and order (Verma and Daniell 2007; Jansen and Ruhlman 2012). In fact, chloroplast genomes have been successfully used as a super barcode to identify numerous species and individuals (Doorduin et al., 2011; Kane et al., 2012; Chen et al., 2019; Wu et al., 2020). The phylogenetic trees constructed in this study demonstrated that complete chloroplast genome sequences can also be used as a super barcode for the identification of *Bidens* species. *Bidens pilosa* var. *pilosa* and *B. pilosa* var. *radiata* had similar morphological characteristics. In the Flora of China database, *B. pilosa* var. *radiata* and *B. pilosa* var. *pilosa* have been merged into one species named *B. pilosa*, and the Latin name *B. pilosa* var. *radiata* has been listed as a synonym of *B. pilosa*. In this study, the chloroplast genomes of *B. pilosa* var. *pilosa* and *B. pilosa* var. *radiata* were similar to each other. The ML phylogenetic tree showed that *B. pilosa* var. *radiata* and *B. pilosa* var. *pilosa* clustered together with a bootstrap value of 100%. Therefore, the analysis results of this study provided support for the merging of *B. pilosa* var. *pilosa* and *B. pilosa* var. *radiata* into the same species.

## Conclusion

In this study, 12 chloroplast and eight mitochondrial genomes of five species and one variety of *Bidens* were analysed. Then, identification and phylogenetic analysis were performed by constructing phylogenetic trees on the basis of the complete chloroplast genomes, complete mitochondrial genomes, common protein-coding genes shared by the chloroplast genomes, common protein-coding genes shared by the mitochondrial genomes, and seven selected highly variable regions. The results of phylogenetic trees based on the complete chloroplast genomes and *trnS-GGA-rps4* showed that different samples of the same species clustered together. This work indicated that complete chloroplast genomes could be used as a super barcode to authenticate *Bidens* species accurately, and the screened highly variable region *trnS-GGA-rps4* could be used as a potential specific barcode to identify *Bidens* species.

## Data Availability

The original contributions presented in the study are publicly available. This data can be found here: The 12 chloroplast genomes and eight mitochondrial genomes of the six *Bidens* species assembled in this study were deposited in GenBank with the accession numbers MW551948-MW551958, MW331585 (chloroplast genomes), and MW838187-MW838194 (mitochondrial genomes).
