# Supplementary material for: Identification of Medicinal Bidens Plants for Quality Control Based on Organelle Genomes
Source: Front Pharmacol. 2022 Feb 14;13:842131. doi: 10.3389/fphar.2022.842131 (PMC8887618; doi:10.3389/fphar.2022.842131)
Supplement: Supplementary file 1 [file DataSheet1.docx]

Supplementary Material

**1 Supplementary Tables and Figures**

**1.1 Supplementary Tables**

**Supplementary Table S1** Information of voucher specimens.

| No. | Species | Specimen ID numbers | Collecting Locations |
| --- | --- | --- | --- |
| 1 | *B. biternata* | Y17107MT01 | Lichuan, Hubei Province, China |
| 2 | *B. biternata* | Y17113MT01 | Xian, Shanxi Province, China |
| 3 | *B. biternata* | Y18108MT01 | Hangzhou, Zhejiang Province, China |
| 4 | *B. bipinnata* | Y17103MT01 | Baoding, Hebei Province, China |
| 5 | *B. bipinnata* | Y17116MT01 | Haidian District, Beijing, China |
| 6 | *B. bipinnata* | Y18102MT01 | Taian, Shandong Province, China |
| 7 | *B. bipinnata* | Y18090MT01 | Xianyang, Shanxi Province, China |
| 8 | *B. pilosa* var. *pilosa*  *B. pilosa* var. *radiata*  *B. pilosa* var. *radiata* | Y17115MT01 | Haidian District, Beijing, China |
| 9 |  | Y17114MT01 | Haidian District, Beijing, China |
| 10 |  | Y17117MT01 | Meizhou, Guangdong Province, China |
| 11 | *B. parviflora* | Y17081MT01 | Haidian District, Beijing, China |
| 12 | *B. tripartita* | Y17102MT01 | Baoding, Hebei Province, China |

**Supplementary Table S2** Chloroplast genome sequencing information of *Bidens* species.

| Sample ID | Insert size (bp) | Raw reads | Raw data (bp) | Raw data Q20(%) | Raw data Q30 (%) | Raw data GC (%) |
| --- | --- | --- | --- | --- | --- | --- |
| Y17107 | 350 | 37907834 | 5686175100 | 96.75 | 92.91 | 37.93 |
| Y17113 | 350 | 49405906 | 7410885900 | 97.64 | 94.5 | 37.64 |
| Y18108 | 350 | 41059018 | 6144092758 | 96.71 | 92.42 | 37.51 |
| Y17103 | 350 | 40575112 | 6086266800 | 96.69 | 92.82 | 37.45 |
| Y17116 | 350 | 41223138 | 6183470700 | 96.99 | 93.3 | 37.88 |
| Y18102 | 350 | 62014686 | 9302202900 | 96.13 | 91.34 | 36.45 |
| Y18090 | 350 | 47648700 | 7147305000 | 97.11 | 91.74 | 37 |
| Y17115 | 350 | 36610188 | 5491528200 | 96.27 | 92.11 | 37.19 |
| Y17114 | 350 | 43127630 | 6469144500 | 96.47 | 92.5 | 37.39 |
| Y17117 | 350 | 39664336 | 5949650400 | 96.59 | 92.54 | 37.35 |
| Y17081 | 350 | 35922832 | 5388424800 | 97.69 | 93.5 | 36.53 |
| Y17102 | 350 | 46475406 | 6971310900 | 97.24 | 93.74 | 38.09 |

**Supplementary Table S3** Mitochondrial genome sequencing information of *Bidens* species.

| Sample ID | mt length (bp) | raw reads | raw data (bp) | mt reads | mt reads ratio (%) | mt genome sequencing depth | reads remapping depth |
| --- | --- | --- | --- | --- | --- | --- | --- |
| Y17107 | 198476 | 37907834 | 5686175100 | 146080 | 0.39 | 102 | 86 |
| Y17113 | 198476 | 49405906 | 7410885900 | 172886 | 0.35 | 76 | 111 |
| Y17103 | 198476 | 40575112 | 6086266800 | 149670 | 0.37 | 73 | 94 |
| Y17116 | 198476 | 41223138 | 6183470700 | 157352 | 0.38 | 101 | 95 |
| Y17115 | 183061 | 36610188 | 5491528200 | 241688 | 0.66 | 194 | 155 |
| Y17117 | 213288 | 39664336 | 5949650400 | 308192 | 0.78 | 217 | 168 |
| Y17081 | 195825 | 35922832 | 5388424800 | 280138 | 0.78 | 208 | 203 |
| Y17102 | 216786 | 46475406 | 6971310900 | 261586 | 0.56 | 188 | 154 |

**Supplementary Table S4** Primers for borders verification in the chloroplast genomes of *Bidens*.

| species | Primer name | Sequence (5' to 3') | Length | Tm | GC% |
| --- | --- | --- | --- | --- | --- |
| *B. tripartita* | IL-F | CAAAAGAAGGGAAATCGGCCA | 21 | 59.1 | 47.6 |
|  | IL-R | AGCTGCTATTGAAGCTCCATC | 21 | 58.2 | 47.6 |
|  | LI-F | TCGTACCTTTATTTACTTCGGCTTT | 25 | 58.8 | 36.0 |
|  | LI-R | AAGAAGGGAAATCGGCCACA | 20 | 59.6 | 50.0 |
|  | IS-F | GTCGTTGTGGTCGGACTCTA | 20 | 59.1 | 55.0 |
|  | IS-R | ATCCAAATGGTTAACCCCGTCT | 22 | 59.7 | 45.5 |
|  | SI-F | TTTTCCATCGCTTTGGCATGT | 21 | 59.4 | 42.9 |
|  | SI-R | CAATTCGGTCGTTGTGGTCG | 20 | 59.8 | 55.0 |
| *B. bipinnata*  *B. biternata*  *B. parviflora* | IL-F | CAAAAGAAGGGAAATCGGCCA | 21 | 59.1 | 47.6 |
|  | IL-R | AGCTCCATCTACAAATGGATAAGAC | 25 | 58.5 | 40.0 |
|  | LI-F | CGGCTTTACTAATCACTAAATTGGC | 25 | 58.8 | 40.0 |
|  | LI-R | AAGAAGGGAAATCGGCCACA | 20 | 59.6 | 50.0 |
|  | IS-F | CAATTCGGTCGTTGTGGTCG | 20 | 59.8 | 55.0 |
|  | IS-R | CAGTCAGTATAGCCTCTTTCGGA | 23 | 59.4 | 47.8 |
|  | SI-F | TTTTCCATCGCTTTGGCATGT | 21 | 59.4 | 42.9 |
|  | SI-R | CAATTCGGTCGTTGTGGTCG | 20 | 59.8 | 55.0 |
| *B. pilosa* var. *pilosa*  *B. pilosa* var. *radiata* | IL-F | CAAAAGAAGGGAAATCGGCCA | 21 | 59.11 | 47.6 |
|  | IL-R | AGCTCCATCTACAAATGGATAAGAC | 25 | 58.5 | 40.0 |
|  | LI-F | CAAAAGAAGGGAAATCGGCCA | 21 | 59.1 | 47.6 |
|  | LI-R | AGCTCCATCTACAAATGGATAAGAC | 25 | 58.5 | 40.0 |
|  | IS-F | CAATTCGGTCGTTGTGGTCG | 20 | 59.8 | 55.0 |
|  | IS-R | CAGTCAGTATAGCCTCTTTCGGA | 23 | 59.4 | 47.8 |
|  | SI-F | CTTTGTTTTCCATCGCTTTGGC | 22 | 59.5 | 45.5 |
|  | SI-R | CAATTCGGTCGTTGTGGTCG | 20 | 59.8 | 55.0 |

LI: primers for border of LSC and IRa; IS: primers for border of IRa and SSC; SI: primers for border of SSC and IRb; IL: primers for border of IRb and LSC

**Supplementary Table S5** Statistics for chloroplast genomes of the *Bidens* species.

| Species | *B. biternata* | *B. biternata* | *B. biternata* | *B. bipinnata* | *B. bipinnata* | *B. bipinnata* |
| --- | --- | --- | --- | --- | --- | --- |
| GenBank accession number | MW551950 | MW551951 | MW551958 | MW551949 | MW551954 | MW551957 |
| Genome size (bp) | 151,487 | 151,487 | 151,487 | 151,486 | 151,476 | 151,488 |
| LSC length (bp) | 83,899 | 83,899 | 83,899 | 83,898 | 83,888 | 83,898 |
| SSC length (bp) | 18,188 | 18,188 | 18,188 | 18,188 | 18,188 | 18,188 |
| IRs length (bp) | 24,700 | 24,700 | 24,700 | 24,700 | 24,700 | 24,701 |
| Total GC content (%) | 37.5 | 37.5 | 37.5 | 37.5 | 37.5 | 37.5 |
| Number of genes | 130 | 130 | 130 | 130 | 130 | 130 |
| Number of protein-coding genes | 85 | 85 | 85 | 85 | 85 | 85 |
| Number of tRNAs | 37 | 37 | 37 | 37 | 37 | 37 |
| Number of rRNAs | 8 | 8 | 8 | 8 | 8 | 8 |
| Number of repeat genes | 17 | 17 | 17 | 17 | 17 | 17 |
| Number of pseudogenes | 2 | 2 | 2 | 2 | 2 | 2 |
| Species | *B. bipinnata* | *B. pilosa* var. *pilosa* | *B. pilosa* var. *radiata* | *B. pilosa* var. *radiata* | *B. parviflora* | *B. tripartita* |
| GenBank number | MW551956 | MW551953 | MW551952 | MW551955 | MW551948 | MW331585 |
| Genome size (bp) | 151,485 | 151,496 | 151,503 | 151,635 | 151,314 | 150,489 |
| LSC length (bp) | 83,897 | 83,731 | 83,739 | 83,892 | 83,739 | 83,499 |
| SSC length (bp) | 18,188 | 18,399 | 18,398 | 18,439 | 18,267 | 17,628 |
| IRs length (bp) | 24,700 | 24,683 | 24,683 | 24,652 | 24,654 | 24,681 |
| Total GC content (%) | 37.5 | 37.5 | 37.5 | 37.5 | 37.5 | 37.5 |
| Number of genes | 130 | 130 | 130 | 130 | 130 | 130 |
| Number of protein-coding genes | 85 | 85 | 85 | 85 | 85 | 85 |
| Number of tRNAs | 37 | 37 | 37 | 37 | 37 | 37 |
| Number of rRNAs | 8 | 8 | 8 | 8 | 8 | 8 |
| Number of repeat genes | 17 | 17 | 17 | 17 | 17 | 17 |
| Number of pseudogenes | 2 | 2 | 2 | 2 | 2 | 2 |

**Supplementary Table S6** Genetic composition and types of chloroplast genomes of the *Bidens* species.

| No. | Group of genes | Gene names | Number |
| --- | --- | --- | --- |
| 1 | Photosystem I | *psaA, psaB, psaC, psaI, psaJ* | 5 |
| 2 | Photosystem II | *psbA, psbB, psbC, psbD, psbE, psbF, psbH, psbI, psbJ, psbK, psbL, psbM, psbN, psbT, psbZ* | 15 |
| 3 | Cytochrome b/f complex | *petA, petB*, petD*, petG, petL, petN* | 6 |
| 4 | ATP synthase | *atpA, atpB, atpE, atpF*, atpH, atpI* | 6 |
| 5 | NADH dehydrogenase | *ndhA*, ndhB** (×2)*, ndhC, ndhD, ndhE, ndhF, ndhG, ndhH, ndhI, ndhJ, ndhK* | 12(1) |
| 6 | RubisCO large subunit | *rbcL* | 1 |
| 7 | RNA polymerase | *rpoA, rpoB, rpoC1*, rpoC2* | 4 |
| 8 | Ribosomal proteins (SSU) | *rps2, rps3, rps4, rps7*(×2)*, rps8, rps11, rps12** (×2)*, rps14, rps15, rps16*, rps18, rps19* | 14(2) |
| 9 | Ribosomal proteins (LSU) | *rpl2**(×2)*, rpl14, rpl16*, rpl20, rpl22, rpl23*(×2)*, rpl32, rpl33, rpl36* | 11(2) |
| 10 | Proteins of unknown function | *ycf1, ycf2(*×2*), ycf3**, ycf4* | 5(1) |
| 11 | Transfer RNAs | 37 *tRNAs* (8 contain an intron, 7 in the IRs) | 37(7) |
| 12 | Ribosomal RNAs | *rrn4.5*(×2)*, rrn5*(×2)*, rrn16*(×2)*, rrn23*(×2) | 8(4) |
| 13 | Other genes | *accD, clpP**, matK, ccsA, cemA, infA* | 6 |

* gene contains one intron; ** gene contains two introns; (×2) indicates the number of the repeat unit is 2.

**Supplementary Table S7** Statistics for mitochondrial genomes of the *Bidens* species.

| Species | *B. biternata*/*B. bipinnata* | *B. pilosa* var. *pilosa* | *B. pilosa* var. *radiata* | *B. parviflora* | *B. tripartita* |
| --- | --- | --- | --- | --- | --- |
| Genbank accession number | MW838190/MW838191/  MW838189/MW838193 | MW838192 | MW838194 | MW838187 | MW838188 |
| Genome length (bp) | 198,476 | 183,061 | 213,288 | 195,825 | 216,786 |
| GC (%) | 45.4 | 45.8 | 45.7 | 45.7 | 45.5 |
| Total gene length (bp) | 52,118 | 49,139 | 52,029 | 56,045 | 54,877 |
| Number of total gene | 83 | 81 | 87 | 90 | 82 |
| CDS | 30 | 30 | 32 | 33 | 29 |
| CDS length (bp) | 28,698 | 29,394 | 31,464 | 31,923 | 27,696 |
| tRNA | 18 | 20 | 19 | 19 | 19 |
| tRNA length (bp) | 1,373 | 1,520 | 1,406 | 1,428 | 1,439 |
| rRNA | 3 | 3 | 3 | 4 | 6 |
| rRNA length (bp) | 5,694 | 5,697 | 5,695 | 5,549 | 11,390 |
| ORF | 32 | 28 | 33 | 34 | 28 |
| ORF length (bp) | 16,353 | 12,528 | 13,464 | 17,145 | 14,352 |

**Supplementary Table S8** Genetic composition and types of mitochondrial genomes of the *Bidens* species.

| Gene Type | Species | | | | |
| --- | --- | --- | --- | --- | --- |
|  | *B. biternata*/*B. bipinnata* | *B. pilosa* var. *pilosa* | *B. pilosa* var. *radiata* | *B. parviflora* | *B. tripartita* |
| complex I (NADH dehydrogenase) | *nad1, nad2, nad3, nad4, nad4L, nad5, nad6, nad7, nad9* | *nad1, nad2, nad3, nad4, nad4L, nad5, nad6, nad7, nad9* | *nad1, nad2, nad3, nad4, nad4L, nad5, nad6, nad7, nad9* | *nad1, nad2, nad3, nad4, nad4L, nad5, nad6, nad7, nad9* | *nad1, nad2, nad3, nad4, nad4L, nad5, nad6, nad7, nad9* |
| complex III (ubichinol cytochrome c reductase) | *cob* | *cob* | *cob* | *cob* | *cob* |
| complex IV (cytochrome c oxidase) | *cox1, cox2, cox3* | *cox1, cox2, cox3* | *cox1, cox2, cox3* | *cox1, cox2, cox3* | *cox1, cox2, cox3* |
| complex V (ATP synthase) | *atp1, atp4, atp6, atp8, atp9* | *atp1, atp4, atp6, atp8, atp9* | *atp1*(×2)*, atp4, atp6, atp8, atp9* | *atp1*(×2)*, atp4, atp6, atp8*(×2)*, atp9* | *atp1, atp4, atp6, atp8, atp9* |
| ribosomal proteins (SSU) | *rps3, rps4, rps12, rps13* | *rps3, rps4, rps12, rps13* | *rps3, rps4*(×2)*, rps12, rps13* | *rps3, rps4, rps12, rps13* | *rps3, rps12, rps13* |
| ribosomal proteins (LSU) | *rpl5, rpl10* | *rpl5, rpl10* | *rpl5, rpl10* | *rpl5, rpl10* | *rpl5, rpl10* |
| maturases | *matR* | *matR* | *matR* | *matR* | *matR* |
| other genes | *ccmB, ccmC, ccmFc, ccmFn, tatC* | *ccmB, ccmC, ccmFc, ccmFn, tatC* | *ccmB, ccmC, ccmFc, ccmFn, tatC* | *ccmB, ccmC, ccmFc, ccmFn, tatC* | *ccmB, ccmC, ccmFc, ccmFn, tatC* |
| Number of ORFs | 32 | 28 | 33 | 34 | 28 |
| Number of transfer RNAs | 18 | 20 | 19 | 19 | 19 |
| ribosomal RNAs | *rrn5, rrnL, rrnS* | *rrn5, rrnL, rrnS* | *rrn5, rrnL, rrnS* | *rrn5*(×2)*, rrnL, rrnS* | *rrn5*(×2)*, rrnL*(×2)*, rrnS*(×2) |

(×2) indicates the number of the repeat unit is 2.

**Supplementary Table S9** SSRs in the chloroplast genomes of the *Bidens* species.

| SSR type | Repeats | Number | | | | | | |
| --- | --- | --- | --- | --- | --- | --- | --- | --- |
|  |  | *B. biternata* | *B. bipinnata* | *B. pilosa* var. *pilosa* | *B. pilosa* var. *radiata* | | *B. parviflora* | *B. tripartita* |
|  |  | MW551950/MW551951/  MW551958 | MW551949/MW551954/  MW551956/MW551957 | MW551953 | MW551952 | MW551955 | MW551948 | MW331585 |
| Mono | A/T | 42 | 41 | 36 | 37 | 41 | 33 | 50 |
|  | C/G | 1 | 1 | 3 | 3 | 1 | 1 | 2 |
| Di | AG/CT | 1 | 1 | 1 | 1 | 1 | 1 | 0 |
|  | AT/AT | 5 | 5 | 6 | 6 | 7 | 6 | 6 |
| Tri | AAC/GTT | 0 | 0 | 0 | 0 | 0 | 1 | 0 |
|  | AAG/CTT | 3 | 3 | 3 | 3 | 3 | 2 | 3 |
|  | AAT/ATT | 1 | 1 | 1 | 1 | 2 | 1 | 0 |
| Tetra | AAAC/GTTT | 0 | 0 | 0 | 0 | 0 | 1 | 1 |
|  | AAAG/CTTT | 2 | 2 | 2 | 2 | 2 | 1 | 2 |
|  | AAAT/ATTT | 4 | 4 | 4 | 4 | 4 | 6 | 4 |
|  | AATC/ATTG | 1 | 1 | 1 | 1 | 1 | 1 | 1 |
|  | AGAT/ATCT | 1 | 1 | 1 | 1 | 1 | 2 | 2 |
| Penta | AAGAT/ATCTT | 0 | 0 | 1 | 1 | 1 | 0 | 0 |
| Hexa | AAATTG/AATTTC | 1 | 1 | 1 | 1 | 1 | 0 | 0 |
| Total |  | 62 | 61 | 60 | 61 | 65 | 56 | 71 |

**Supplementary Table S10** SSRs in the mitochondrial genomes of the *Bidens* species.

| SSR type | Repeats | Species | | | | | |
| --- | --- | --- | --- | --- | --- | --- | --- |
|  |  | *B. biternata* | *B. bipinnata* | *B. pilosa* var. *pilosa* | *B. pilosa* var. *radiata* | *B. parviflora* | *B. tripartita* |
|  |  | MW838190/MW838191 | MW838189/MW838193 | MW838192 | MW838194 | MW838187 | MW838188 |
| Mono | A/T | 4 | 4 | 6 | 6 | 6 | 8 |
|  | C/G | 2 | 2 | 4 | 5 | 1 | 3 |
| Di | AG/CT | 4 | 4 | 3 | 4 | 2 | 6 |
|  | AT/AT | 5 | 5 | 6 | 8 | 6 | 5 |
| Tri | AAG/CTT | 6 | 6 | 3 | 6 | 4 | 4 |
|  | AAT/ATT | 3 | 3 | 2 | 2 | 3 | 1 |
|  | AGC/CTG | 0 | 0 | 0 | 0 | 1 | 1 |
| Tetra | AAAC/GTTT | 1 | 1 | 0 | 0 | 1 | 0 |
|  | AAAG/CTTT | 6 | 6 | 7 | 7 | 5 | 5 |
|  | AAAT/ATTT | 1 | 1 | 1 | 1 | 1 | 2 |
|  | AACG/CGTT | 1 | 1 | 1 | 1 | 1 | 0 |
|  | AAGC/CTTG | 1 | 1 | 1 | 2 | 1 | 1 |
|  | AAGG/CCTT | 1 | 1 | 1 | 1 | 1 | 2 |
|  | AAGT/ACTT | 1 | 1 | 0 | 1 | 1 | 0 |
|  | AATC/ATTG | 2 | 2 | 1 | 3 | 2 | 2 |
|  | AATG/ATTC | 2 | 2 | 2 | 2 | 3 | 3 |
|  | ACAG/CTGT | 0 | 0 | 0 | 0 | 0 | 1 |
|  | ACAT/ATGT | 1 | 1 | 1 | 1 | 1 | 0 |
|  | ACCG/CGGT | 3 | 3 | 3 | 3 | 3 | 2 |
|  | AGCC/CTGG | 1 | 1 | 1 | 1 | 1 | 0 |
|  | CCCG/CGGG | 3 | 3 | 3 | 3 | 4 | 4 |
|  | CCGG/CCGG | 2 | 2 | 2 | 2 | 2 | 2 |
| Penta | AAAAG/CTTTT | 1 | 1 | 1 | 1 | 1 | 1 |
|  | AACTT/AAGTT | 0 | 0 | 0 | 0 | 0 | 2 |
|  | ACTAG/AGTCT | 1 | 1 | 1 | 1 | 1 | 1 |
| Hexa | AAAATC/ATTTTG | 1 | 1 | 0 | 1 | 0 | 0 |
|  | AAGATG/ATCTTC | 1 | 1 | 0 | 0 | 1 | 0 |
|  | total | 54 | 54 | 50 | 62 | 53 | 56 |

**Supplementary Table S11** Repeat sequences in chloroplast genomes of the *Bidens* species.

| Species | Genbank accession number | Complement | Forward | | | | | Palindrome | | | | | Reverse |
| --- | --- | --- | --- | --- | --- | --- | --- | --- | --- | --- | --- | --- | --- |
|  |  | 30-39 | 30-39 | 40-49 | 50-59 | 60-69 | ≥70 | 30-39 | 40-49 | 50-59 | 60-69 | ≥70 | 30-39 |
| *B. biternata* | MW551950/MW551951/MW551958 | 2 | 14 | 3 |  |  | 2 | 13 | 5 |  |  | 2 | 5 |
| *B. bipinnata* | MW551949/MW551954/MW551957/MW551956 | 2 | 14 | 3 |  |  | 2 | 13 | 5 |  |  | 2 | 5 |
| *B. pilosa* var. *pilosa* | MW551953 | 3 | 36 | 5 | 1 | 3 | 4 | 24 | 10 |  | 2 | 4 | 13 |
| *B. pilosa* var. *radiata* | MW551952 | 5 | 40 | 5 | 1 | 3 | 4 | 25 | 10 |  | 2 | 4 | 15 |
| *B. pilosa* var. *radiata* | MW551955 | 6 | 19 | 4 | 1 | 2 | 2 | 19 | 5 |  | 3 | 2 | 10 |
| *B. parviflora* | MW551948 | 1 | 19 | 3 |  |  | 2 | 14 | 6 |  |  | 2 | 3 |
| *B. tripartita* | MW331585 | 3 | 27 | 5 | 2 |  | 2 | 21 | 10 | 2 |  | 2 | 12 |

**Supplementary Table S12** Repeat sequences in mitochondrial genomes of the *Bidens* species.

| Species | Genbank accession number | Complement | Forward | | | | Palindrome | | | | Reverse |
| --- | --- | --- | --- | --- | --- | --- | --- | --- | --- | --- | --- |
|  |  | 30-39 | 30-39 | 40-99 | 100-999 | ≥1000 | 30-39 | 40-99 | 100-999 | ≥1000 | 30-39 |
| *B. biternata* | MW838190/MW838191 | 2 | 37 | 9 | 8 | 0 | 32 | 11 | 8 | 2 | 6 |
| *B. bipinnata* | MW838189/MW838193 | 2 | 37 | 9 | 8 | 0 | 32 | 11 | 8 | 2 | 6 |
| *B. pilosa* var. *pilosa* | MW838192 | 2 | 30 | 20 | 7 | 1 | 44 | 12 | 10 | 2 | 4 |
| *B. pilosa* var. *radiata* | MW838194 | 0 | 59 | 39 | 7 | 1 | 40 | 14 | 9 | 0 | 3 |
| *B. parviflora* | MW838187 | 0 | 45 | 13 | 4 | 1 | 37 | 19 | 8 | 2 | 2 |
| *B. tripartita* | MW838188 | 0 | 57 | 38 | 5 | 8 | 40 | 19 | 12 | 2 | 0 |

**1.2 Supplementary Figures**


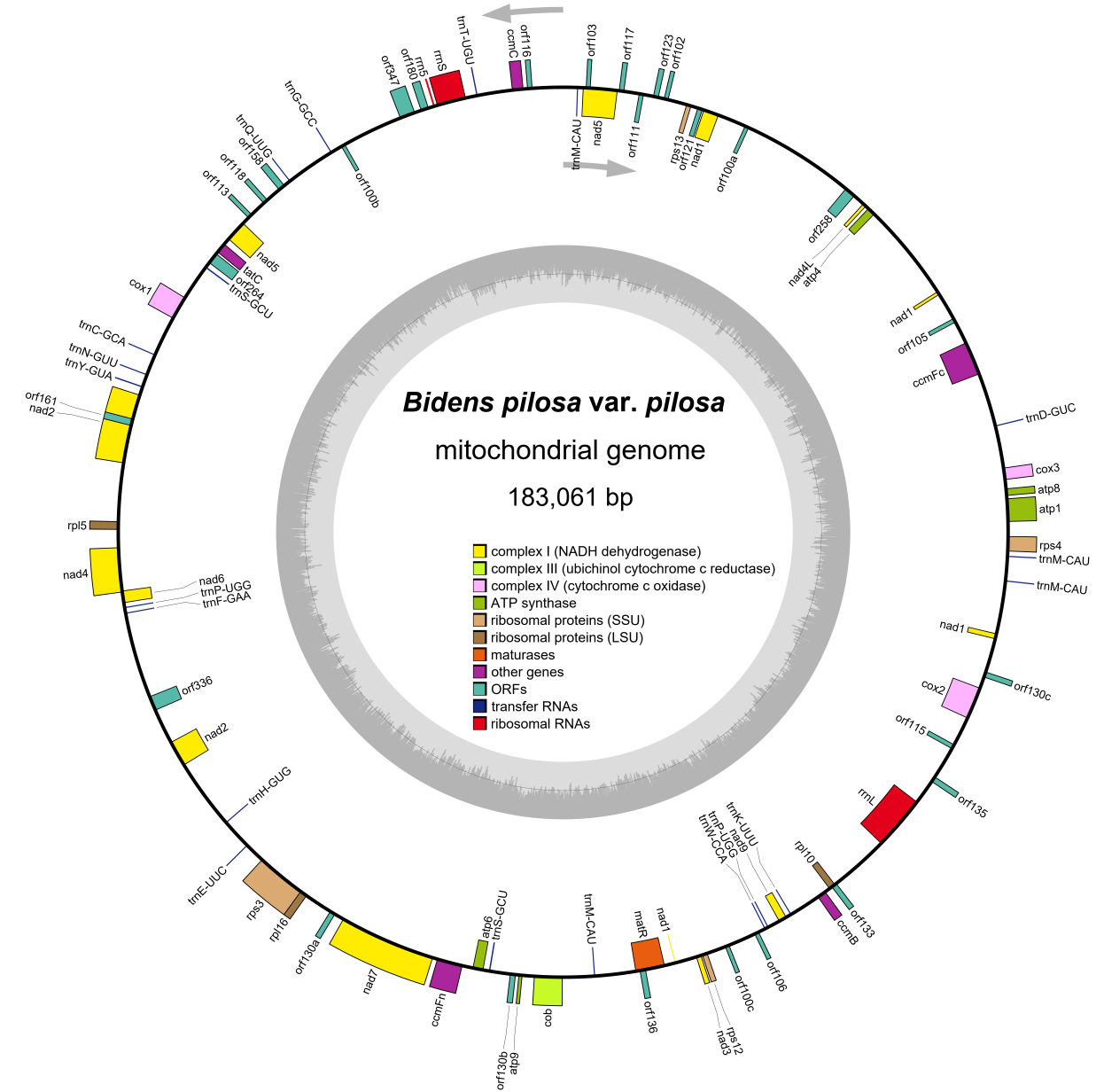


**Supplementary Figure S1-1** Mitochondrial genome map of *B. pilosa* var. *pilosa*.


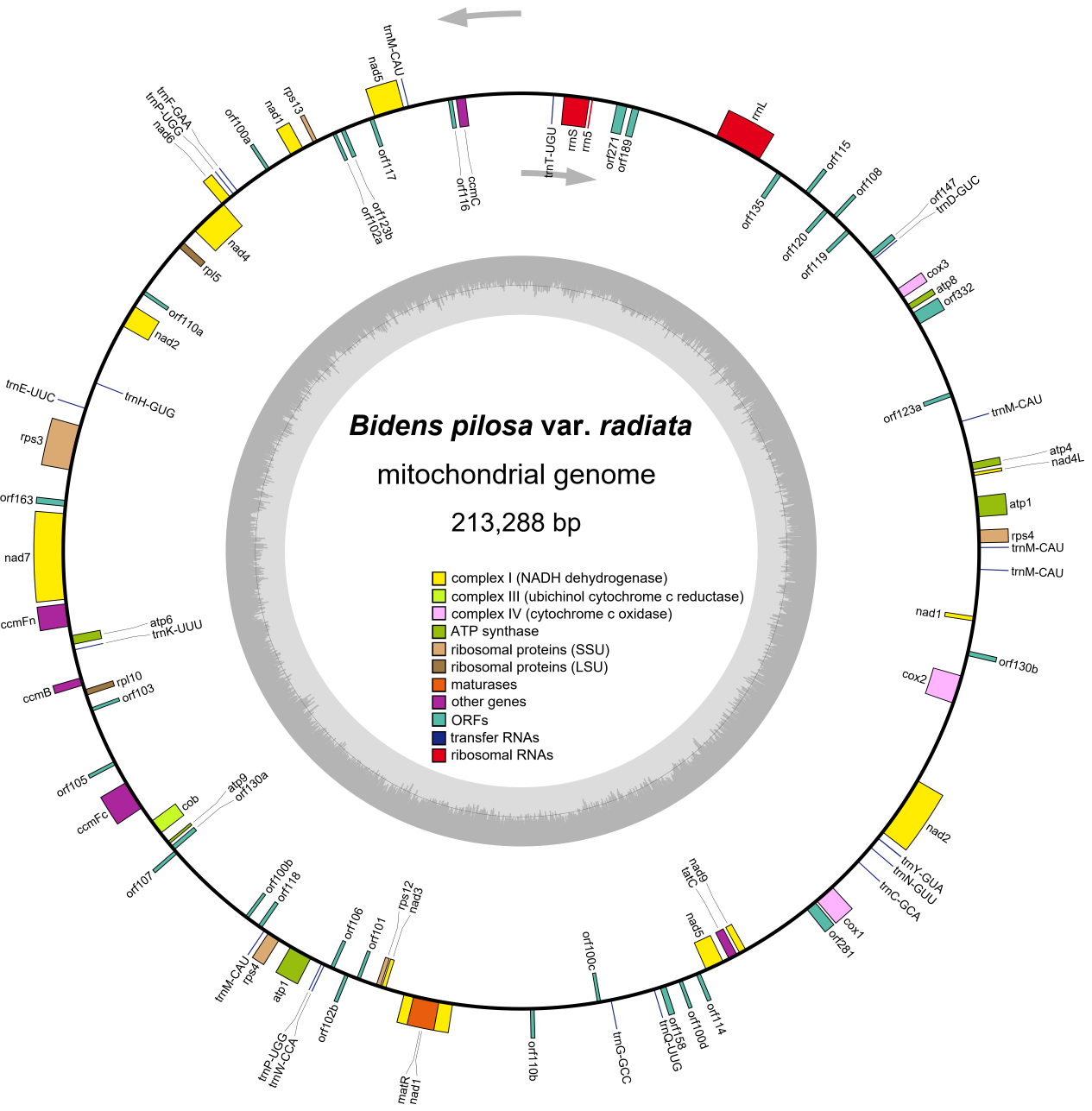


**Supplementary Figure S1-2** Mitochondrial genome map of *B. pilosa* var. *radiata*.


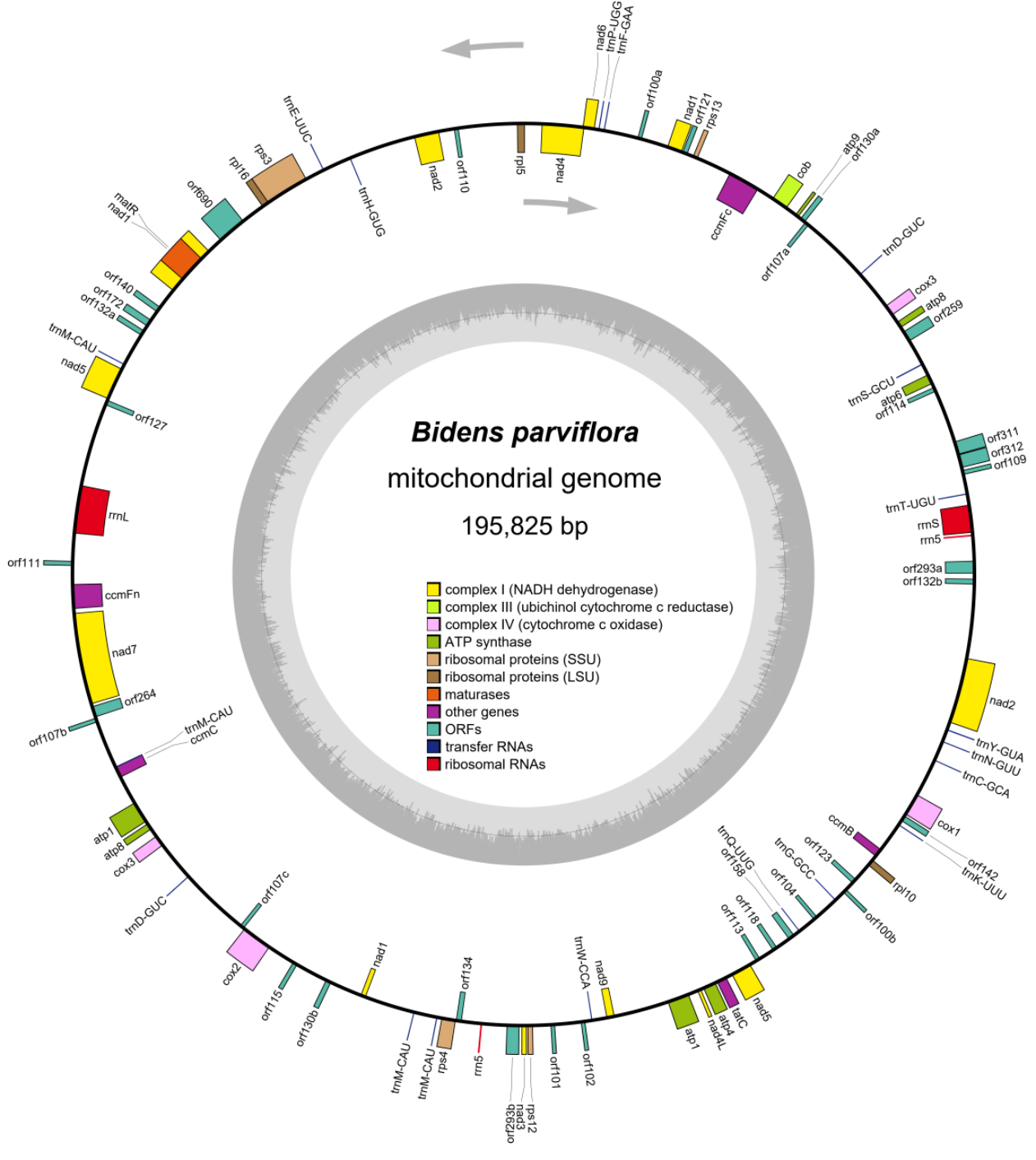


**Supplementary Figure S1-3** Mitochondrial genome map of *B. parviflora*.


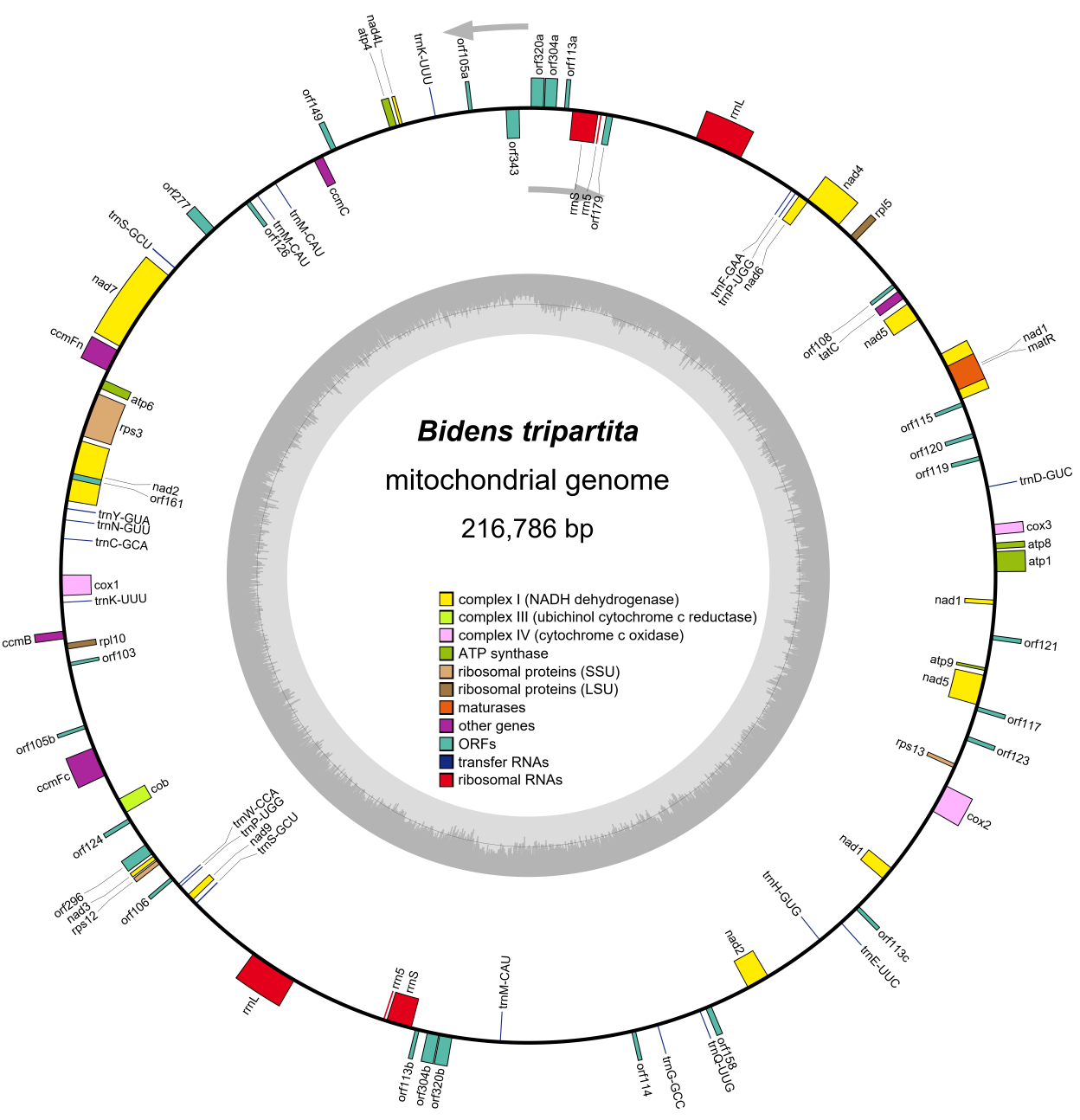


**Supplementary Figure S1-4** Mitochondrial genome map of *B. tripartita*.


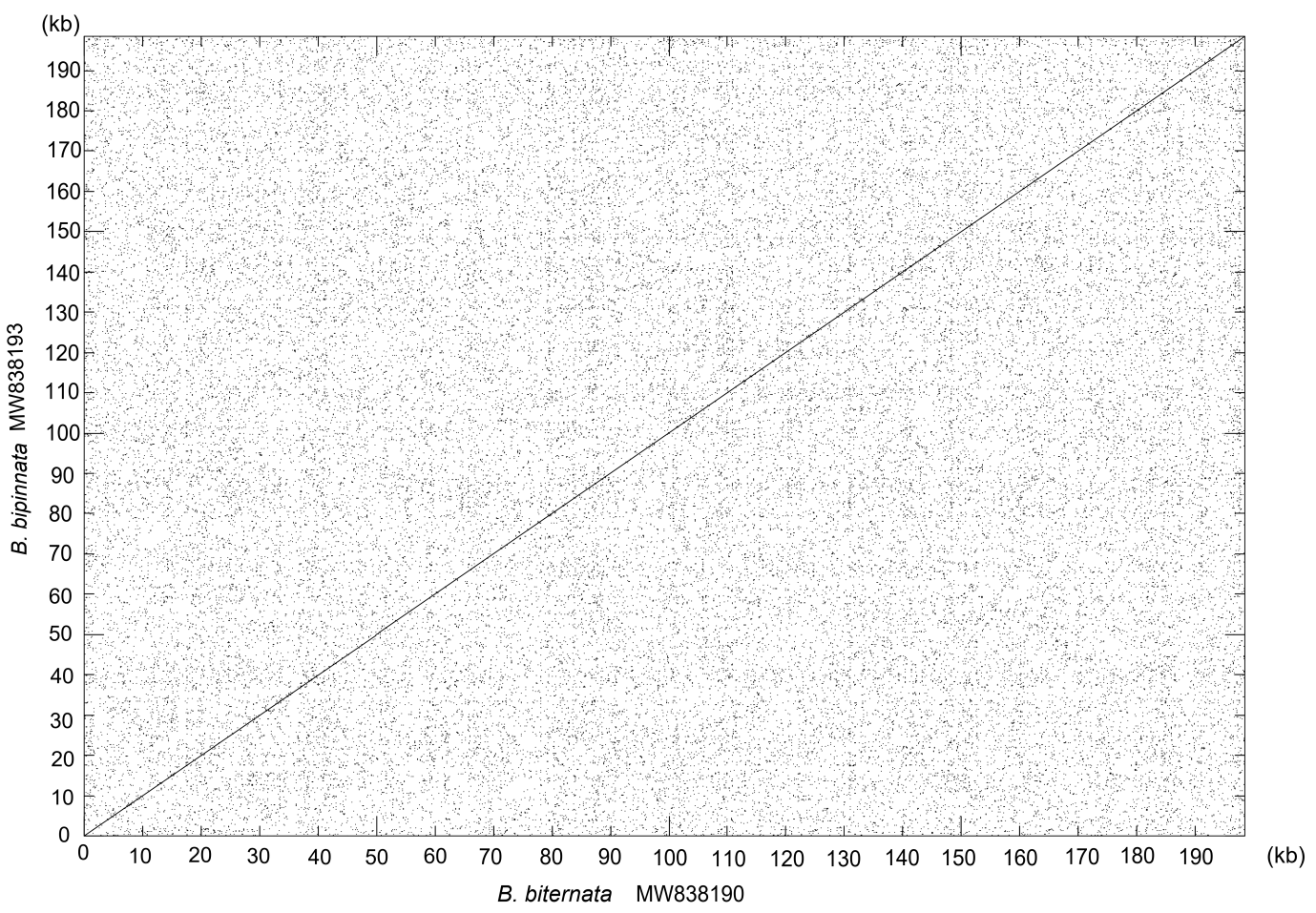


**Supplementary Figure S2** Dot-plot alignment of *B. biternata* and *B. bipinnata* mitochondrial genome sequences.


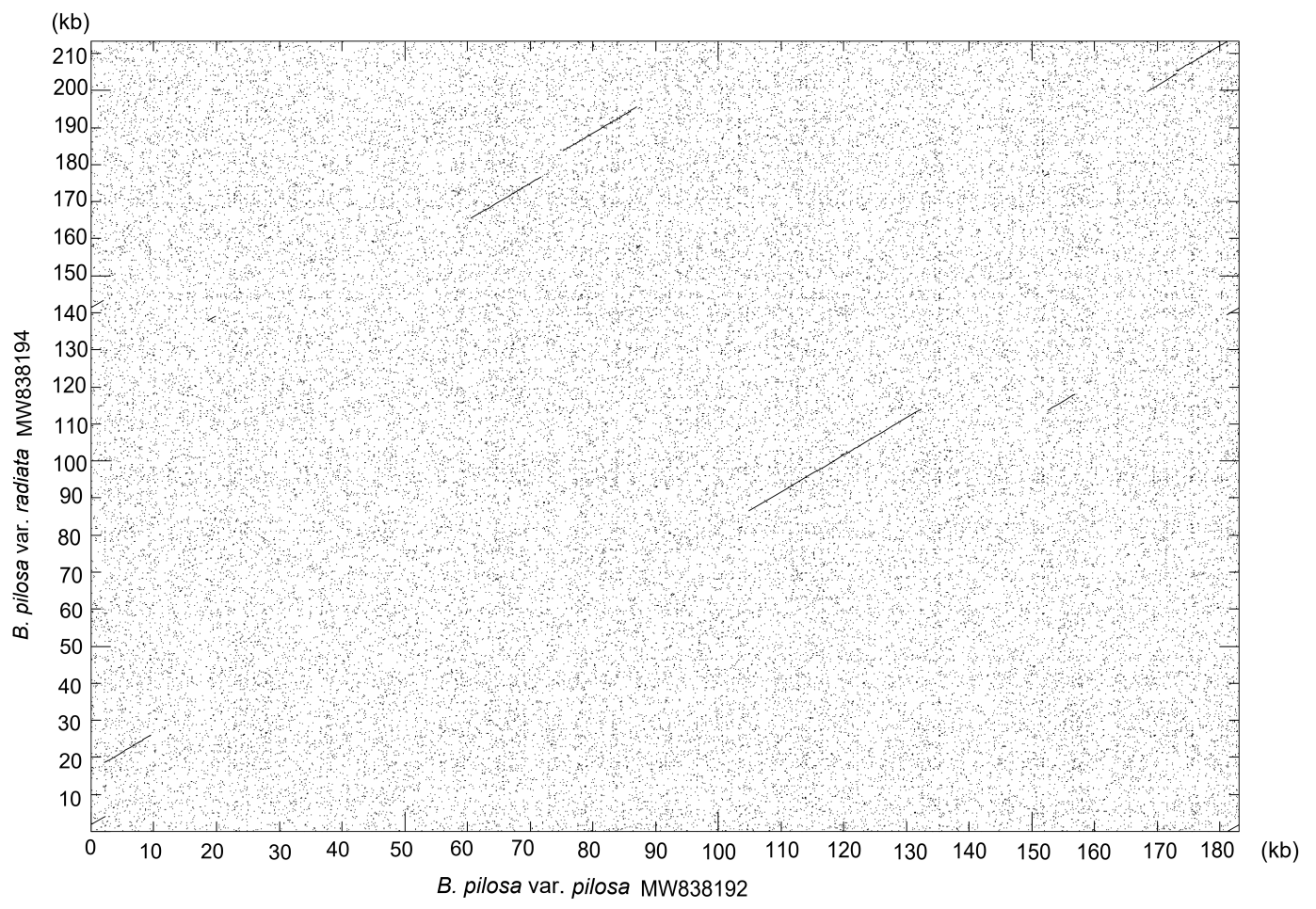


**Supplementary Figure S3** Dot-plot alignment of *B. pilosa* var. *pilosa* and *B. pilosa* var. *radiata* mitochondrial genome sequences.


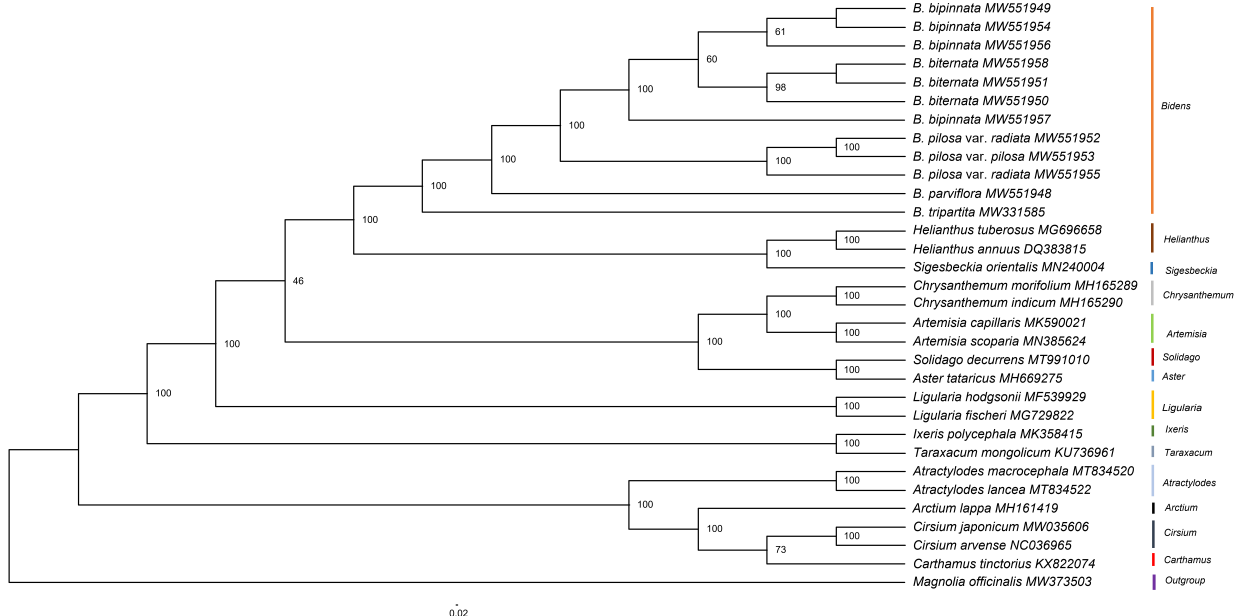


**Supplementary Figure S4** Phylogenetic tree of Asteraceae species inferred by maximum likelihood (ML) analyses based on common protein-coding genes of chloroplast genomes. Numbers at nodes are values for bootstrap support.


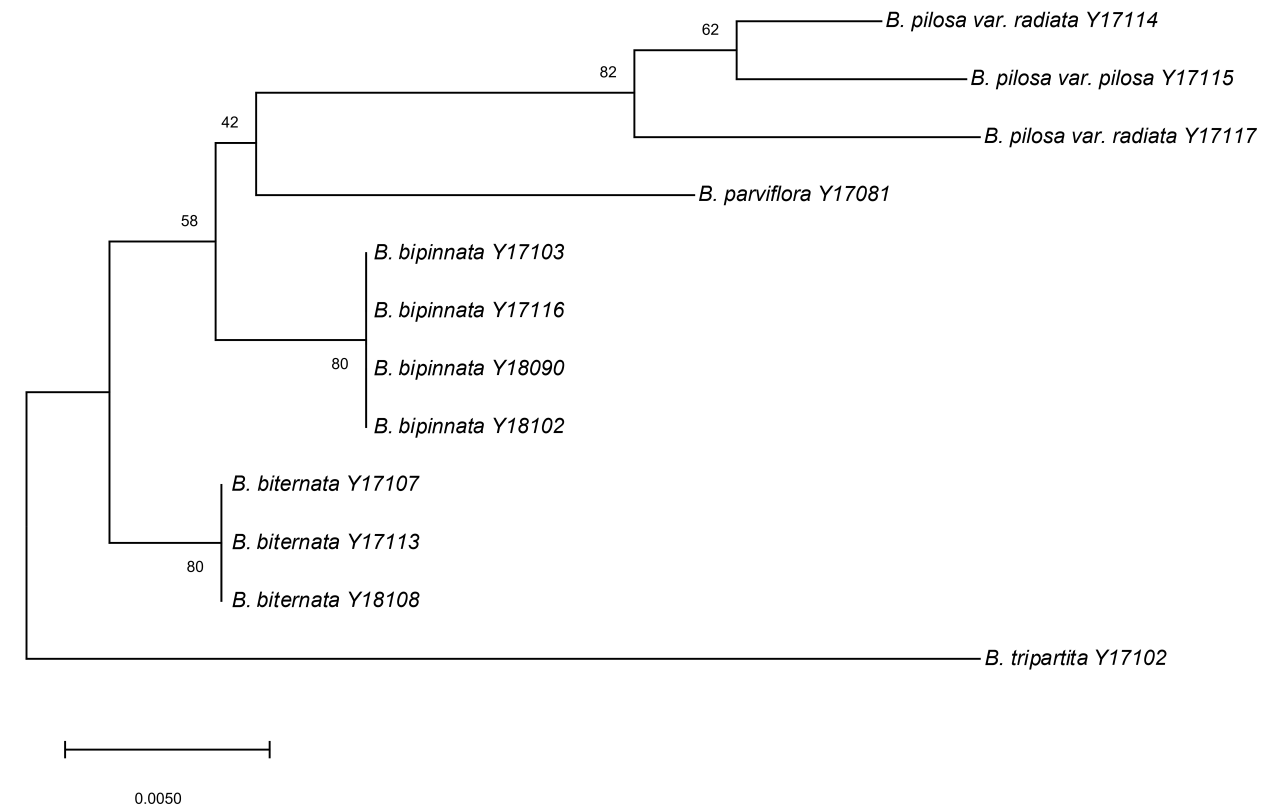


**Supplementary Figure S5** Phylogenetic tree constructed using NJ method based on *trnS-GGA-rps4* of chloroplast genomes of *Bidens* species.


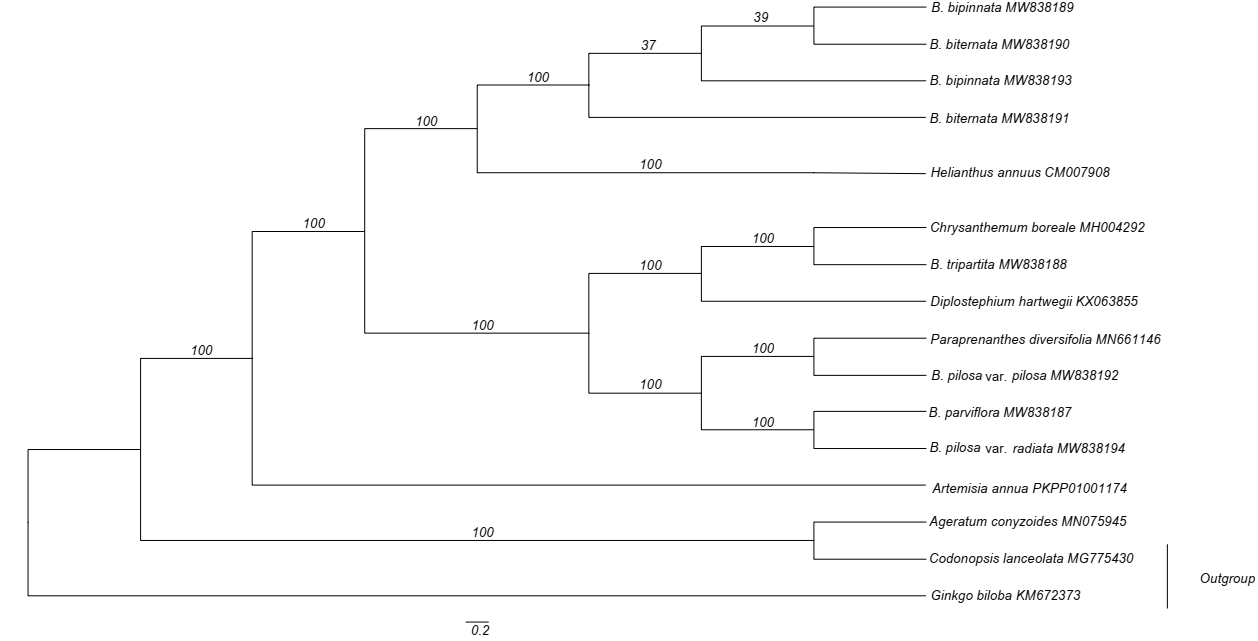


**Supplementary Figure S6** Phylogenetic tree constructed using ML method based on the complete mitochondrial genome sequences of Asteraceae species. Numbers at nodes are values for bootstrap support.


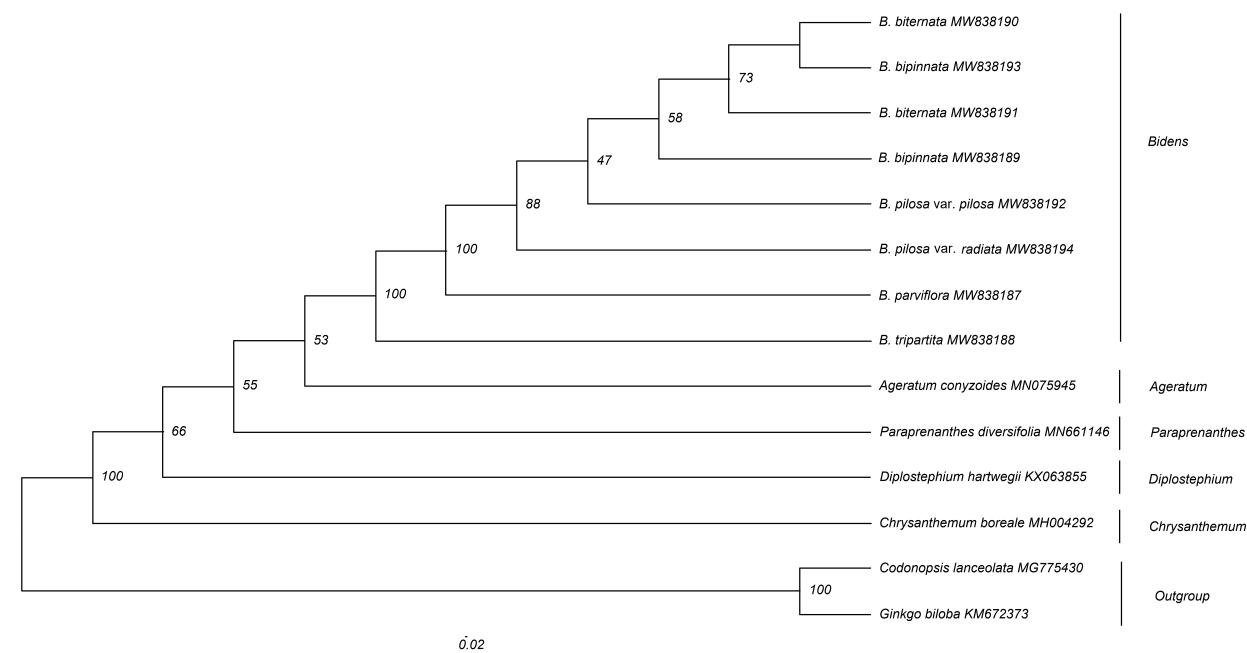


**Supplementary Figure S7** Phylogenetic tree of Asteraceae species inferred by ML analyses based on common protein-coding genes of mitochondrial genomes. Numbers at nodes are values for bootstrap support.


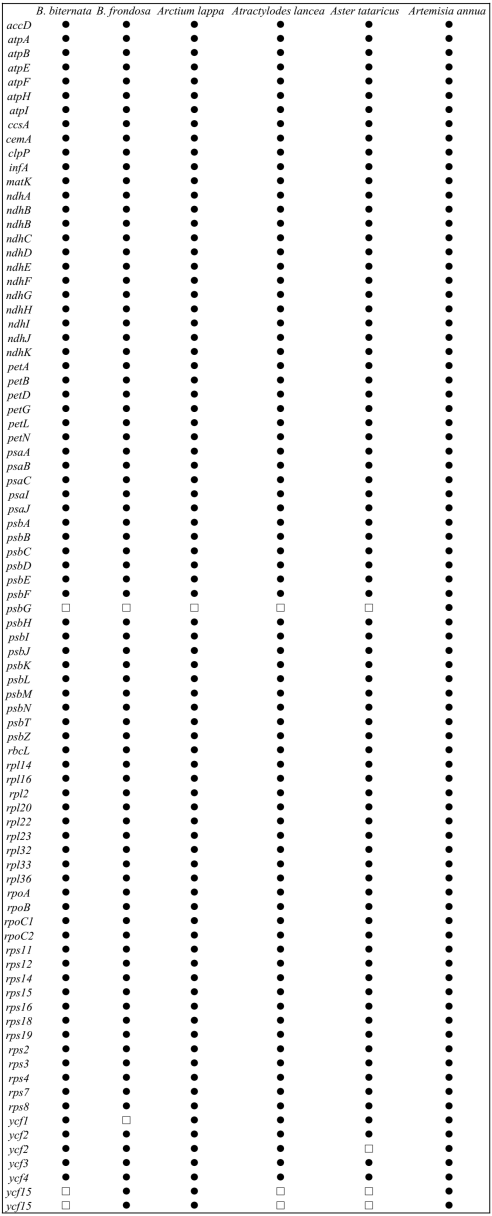


**Supplementary Figure S8** Comparative analysis of CDs gene in 11 medicinal plants of Asteraceae. ● indicates the existence of this gene; □ indicates that the gene is missing. The gene composition of the *Bidens* medicinal plants is the same, so it is represented by *B. biternate.*


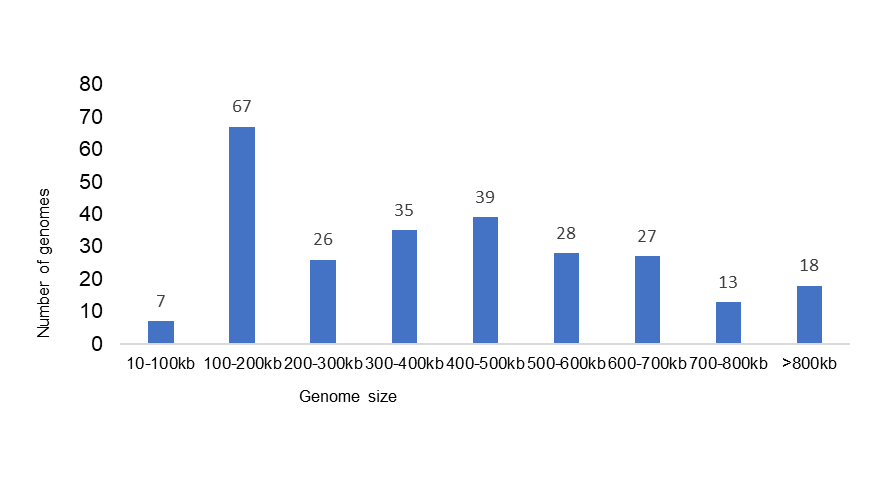


**Supplementary Figure S9** Size of mitochondrial genomes of 260 land plants reported in NCBI.
